# Feature Selection and Hyperparameter Optimization for Machine Learned Classification of 3D Single-Particle Tracking

**DOI:** 10.1021/cbmi.5c00057

**Published:** 2025-08-14

**Authors:** Jagriti Chatterjee, Subhojyoti Chatterjee, Emil Gillett, Nikita Kovalenko, Dongyu Fan, Christy F. Landes

**Affiliations:** † Department of Chemistry, 14589 University of Illinois at Urbana—Champaign, Urbana, Illinois 61801, United States; ‡ Department of Chemistry, Rice University, Houston, Texas 77005, United States; § Department of Chemical and Biomolecular Engineering, University of Illinois at Urbana—Champaign, Urbana, Illinois 61801, United States; ∥ Department of Chemical and Biomolecular Engineering, Rice University, Houston, Texas 77005, United States; ⊥ Department of Electrical and Computer Engineering, University of Illinois at Urbana—Champaign, Urbana, Illinois 61801, United States; # Department of Electrical and Computer Engineering, Rice University, Houston, Texas 77005, United States

**Keywords:** single particle dynamics, diffusion, machine learning, feature selection, hyperparameter optimization

## Abstract

Understanding diffusion in charged and crowded media is crucial for solving a wide range of biological and materials challenges. Classifying diffusion by traditional methods such as mean square displacement in three-dimensional single-particle tracking (3D SPT) is difficult, especially when there are mixed motion types. To address this, we employed machine learning (ML), specifically decision tree algorithms with feature selection, to identify the six most relevant features for accurate characterization of trajectories. This work demonstrates the value of ML in advancing our understanding of heterogeneous transport that occurs in charged and crowded environments, with a broad range of applications.

## Graphical abstract

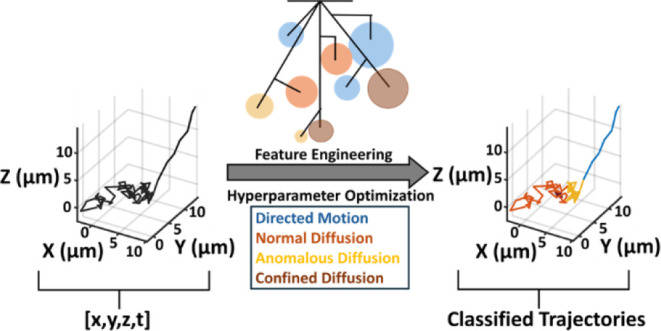

## 1 Introduction

Single-particle tracking (SPT) is an optical method that is broadly used to understand transport dynamics in a wide range of media.^1–5^ Wide-field tracking is performed by imaging particle trajectories on a camera chip, generating time-sequenced trajectories.^6,7^ Unlike methods such as fluorescence recovery after photobleaching (FRAP)^8^ or fluorescence correlation spectroscopy (FCS),^9–13^ SPT does not average the results, which can provide a precise understanding of particle behavior within heterogeneous environments.^14,15^ Some challenges such as localization precision, photoblinking, and low signal-to-noise have been addressed before, but other challenges remain, especially for three-dimensional (3D) SPT and for tracking in charged and crowded environments where heterogeneous transport mechanisms occur interchangeably.^16–20^

Classification of diffusion in trajectories has traditionally relied on methods such as mean square displacement (MSD).^21,22^ MSD classifies motion into directed motion (DM), normal diffusion (ND), anomalous diffusion (AD) and confined diffusion (CD), based on the deviations from linear ND behavior in the MSD plot.^23,24^ However, the reliability of MSD analysis can be compromised by short or noisy trajectories and localization precision in the data,^21,25,26^ and when multiple types of motion are present. Additionally, methods like Hidden Markov Models and moment scaling spectrum (MSS) are available for characterizing different types of motion which can detect not only the differences in particle behavior but also their confinement within a specific time. However, these methods encounter limitations when dealing with short or noisy trajectories, when working with large amounts of trajectory data, or when heterogeneous motion is present.^27–31^

Artificial intelligence (AI) has emerged as a vital tool for managing and analyzing large data sets,^32,33^ with machine learning (ML)^34,35^ and deep learning (DL)^36,37^ increasingly employed in trajectory classification.^17,25–40^ For example, Wagner et al. utilized a random forest (RF) algorithm to classify DM, ND, AD, and CD motions by leveraging nine different trajectory features.^41^ Similarly, Granik et al. implemented convolutional neural networks (CNN), a type of DL algorithm, to classify Brownian motion (BM), fractional Brownian motion (FBM), and continuous time random walk (CTRW) diffusion modes.^42^ Further expanding on these methodologies, Kowaleck et al. introduced a novel approach that compared CNN with other feature-based methods, including RF and gradient boosting (GB), to classify motion types and select relevant features.^43^ Despite the aforesaid advancements, the existing models predominantly treat two-dimensional (2D) SPT data, and there is a need for AI algorithms that treat 3D SPT data in more complex systems.^42,43^

In this study, we address these gaps by implementing ML to classify different types of motion in 3D single particle trajectories. Our approach begins with a thorough feature selection process to identify the most critical features for accurately characterizing particle trajectories.^44–46^ This model is rigorously tested across both simulated scenarios and experimental data sets such as transport in polymer brushes, where high confinement and charge effects introduce complex diffusive behaviors.^47^ By leveraging ML strategies, our study not only enhances the robustness and adaptability of diffusion classification models but also shows the potential to advance our understanding of transport in biological and biomaterials applications.^23^ This approach opens new avenues for deciphering cellular behaviors and pharmacological interactions, highlighting the potential of ML to provide deeper insights into complex biological processes.^48^

## 2 Computational Methodology

The baseline model was developed to classify particle motion into distinct dynamic types using a data set comprising 5000 simulated 3D trajectories, each consisting of 100 frames. These trajectories, generated by Monte Carlo simulations, provided a total of 500,000 data points for training and classification of various types of motion. Figure 1a-e illustrates simulated 3D trajectories for various motion types: DM, ND, AD, CD and Mixed Motion (MM, a combination of DM, ND, CD, and AD). Figure 1f presents a Venn diagram that demonstrates the simulated data set distribution and overlap between different motion categories. The classification workflow in Figure 1g describes the process, starting with input trajectory data in the form of positional coordinates [*x*,*y*,*z*] across 100 frames. Features describing the trajectories are extracted and fed into a decision tree classifier, which predicts the motion type. The workflow for this project is detailed further in Figure S1, providing a step-by-step outline of the simulation, feature extraction, and classification processes.

**Figure 1:**
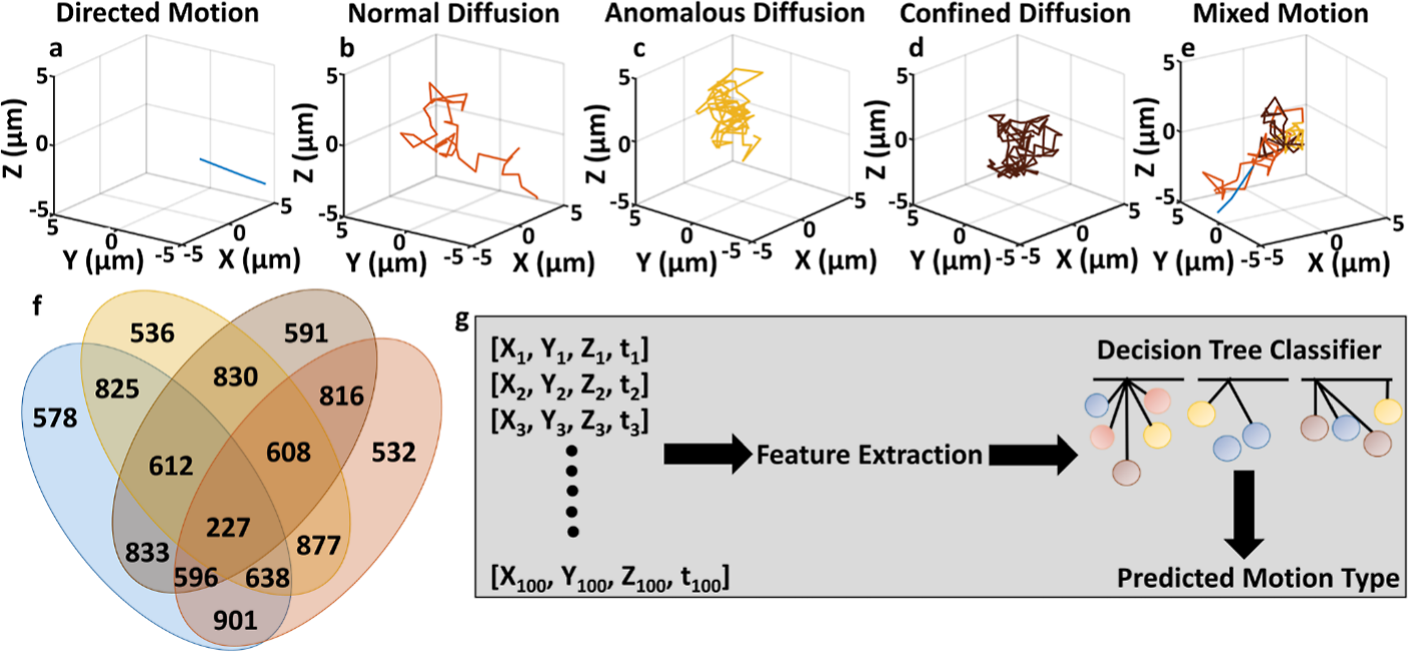
Simulating 3D trajectories for various single-particle motion types. (a) DM, (b) ND, (c) AD, (d) CD, and (e) MM (a combination of DM, ND, AD, and CD), extended over 100 frames. (f) The data set distribution. (g) The workflow pipeline, where features extracted from 3D trajectories are used as input to decision trees.

The trajectories were analyzed using MSD, defined in eq S1, to quantify particle displacement over time. Theoretical models for each motion type (eqs S2–S5) were used to calculate MSD curves, which were then plotted against time to validate the simulated trajectories’ behavior, as shown in Figure S2. These plots confirmed the consistency of the simulations with theoretical expectations, such as the linear time dependence of MSD for ND and the plateauing behavior for CD.

### 2.1 Normal Diffusion

ND describes random movement of particles within a medium in three dimensions.^49^ ND is characterized by a Gaussian distribution of step sizes and a MSD that increases linearly over time. With input time vector *t* and diffusion coefficient *D*, we start by calculating time differentials *dt* between consecutive time points. The step size in each dimension (*x*,*y*,*z*) is drawn from a Gaussian distribution with mean zero and a variance proportional to the product of the diffusion coefficient and the time differential expressed as $\sigma =\sqrt{2D\times dt}$ . Random displacements Δ*x*, Δ*y*, Δ*z* are then generated using μ + σ·randn­(), where μ = 0 and randn­() represents samples from a standard normal distribution. The cumulative sum of these displacements provides the particle’s trajectory in 3D space, which is given by (eq 1)­

1 $$r\left(i\right)=\sum_{k=1}^{i}\Delta r_{k}=\left(\sum_{k=1}^{i}\Delta x_{k},\sum_{k=1}^{i}\Delta y_{k},\sum_{k=1}^{i}\Delta z_{k},\right)$$

where, *r*(*i*) represents the position of the particle at step *i* and Δ*r*_*k*_ = (Δ*x*_*k*_,Δ*y*_*k*_,Δ*z*_*k*_) denotes random displacement vector at step *k*.

### 2.2 Directed Motion

DM is the movement of a single particle toward a specific direction, as we observe in the cellular locomotion.^14^ The deterministic component introduces consistent directionality through a velocity vector *v*, leading to a net drift over time. The particle’s position at any time *t* is described by (eq 2)­

2 $$r\left(t\right)=r_{0}+vt+\Delta r\left(t\right)$$

where *r*_0_ is the initial position, *vt* represents the deterministic motion, and Δ*r*(*t*) is the stochastic displacement due to diffusion, modeled as a Gaussian distribution. The velocity *v* is generated by determining random speed, which is calculated using a coefficient *coef*. This coefficient is uniformly sampled within a defined range [*k*_*dm*1_,*k*_*dm*2_] and scaled by diffusion coefficient *D*. This results in *speed* = *coef*·*D*. The direction of motion is then initialized by generating a random 3D velocity vector from a normal distribution. To introduce angular variability, small angular changes are applied to the velocity vector using a Wiener process.^50^ At each time step, the azimuthal (φ) and polar (θ) angles of the velocity vector are changed by increments Δφ and Δθ, sampled from a Gaussian distribution with variance proportional to the diffusion coefficient. The updated velocity vector is then transformed into Cartesian coordinates.

### 2.3 Anomalous Diffusion

AD occurs when its movement is temporarily hindered, resulting in restricted motion and slower diffusion, as when a protein interacts with a membrane, or with the underlying cytoskeleton.^48^ We employed the Weierstrass–Mandelbrot function^41,51–53^ for the simulation of AD in 3D (eq 3)­

3 $$W\left(t\right)=\sum_{n}\frac{\cos\left(\phi_{n}\right)-\cos\left(\gamma^{n}t^{*}+\phi_{n}\right)}{\gamma^{n\alpha /2}}$$

Anomalous exponent, α < 1, $t^{*}=\frac{2\pi t}{N}$ , γ = √π, ϕ_*n*_ is the random phase that has evenly distributed values between 0 and 2π. According to Saxton’s definition^53^ the sum is taken from *n* = −8 to *n* = 48. To construct a trajectory, we generate *N* displacements, *d*_*i*_ = *W*(*t*) – *W*(*t* – Δ*t*) and sample by choosing *N* > *N*_0_, where, *N*_0_ is the desired number of steps. The displacements for *x*, *y*, *z* directions are generated independently for randomness, which are then scaled to MSD, ⟨*r*_*i*_^2^⟩ = 6*Dt* ^α^ following theoretical anomalous behavior (α < 1).

In this study, we used a model of AD based on the Weierstrass–Mandelbrot function, which captures long-range temporal correlations in particle displacements. This model is well-suited for simulating subdiffusive behavior in obstructed or viscoelastic environments, however, it represents only one form of anomalous transport; other models like CTRW, FBM, and ATTM (annealed transient time model) capture different statistical behaviors.^54^

### 2.4 Confined Diffusion

CD refers to the restricted motion of a particle within a defined spatial region, such as a colloidal particle inside a microfluidic channel.^55^ The first step involves defining the spatial boundary. The radius of confinement *r* is calculated based on the diffusion coefficient *D* and a randomly selected confinement parameter *B*_param_ within specified bounds (eq 4)­

4 $$r=\frac{\sqrt{D}}{B_{param}^{1/3}}$$

Here, *B*_param_ is chosen randomly from a range [*B*_min_,*B*_max_], governing the degree of confinement imposed on the particle’s motion.

The temporal evolution of the simulation is defined by the main time step *dt* (eq 5) which is computed as the difference between elements in the input time vector *t*. For finer resolution a smaller subtime step is defined as *ddt* (eq 6). Where

5 dt=mean(Δt)

6 $$ddt=\frac{dt}{100}$$

We then simulate ND as explained before and ensure that the particle remains within the specified radius of confinement. For this, we calculated the Euclidean distance from the starting point for each step by (eq 7)­

7 $$len=\sqrt{x_{end}^{2}+y_{end}^{2}+z_{end}^{2}}$$

The particle’s position is only accepted if *len* ≤ *r*, otherwise, the step is rejected, and a new step is generated. This ensures that the particle’s motion remains confined within the defined spatial boundary, capturing the diffusive behavior.

### 2.5 Mixed Motion in 3D

MM occurs when a particle transitions between distinct motion types. Using a stochastic framework, we combined motion types within a single trajectory by generating segments of each type with dynamically varying lengths, thereby mimicking complex behaviors observed in natural systems. The simulation parameters for generating MM are provided in Table S1.

### 2.6 Segmentation of Trajectories

A critical step in the model development is the segmentation of continuous trajectories into smaller windows. Windowing helps in localized motion analysis, allowing the model to focus on specific motion patterns within each segment, capturing dynamics in particle behavior over time. The choice of window length is also important to find the balance between granularity and noise: shorter windows captured rapid changes with finer detail but were more prone to noise, while longer windows provided a smoother, more stable overview at the risk of missing minute changes in motion dynamics. The segmentation was achieved using a sliding window approach. Each trajectory was divided into overlapping windows, with each window containing a specified number of frames. For the current work, a window length of 29 frames was chosen to optimize accurate motion classification while minimizing noise without oversmoothing key dynamics. It is important to note that this value would be expected to change depending on the diffusion characteristics and measurement/sampling parameters. The simulated trajectories in our data set also can switch between different types of motion. Each window is treated as an independent sample and labeled according to the simulated motion type at its center frame. This setup allows the model to learn local motion characteristics and effectively capture dynamic switching behavior across a trajectory. Classification accuracy is calculated as the fraction of windows with correctly predicted labels at their centers, providing a detailed assessment of model performance on mixed-motion data.

### 2.7 Feature Extraction

Following trajectory segmentation, to capture the characteristics of each trajectory, we generated and extracted 14 distinct features: alpha, angular Gaussianity index, asymmetry, avg. MSD ratio, efficiency, fractal dimension, Gaussianity, jump length, kurtosis, maximal excursion, mean maximal excursion, straightness, trappedness, and velocity autocorrelation from the segmented trajectories. Each feature is detailed in Section 2—Trajectory Feature Extraction of the Supporting Information. The feature extraction step was crucial for understanding complex motion behaviors into data that could be used by the classifier.

### 2.8 Random Forest Model Development

The extracted features were used to train a RF classifier, implemented using the *TreeBagger* function in MATLAB. RF was chosen for its ability to handle complex, nonlinear relationships in high-dimensional data and its resilience against overfitting. The data set was divided into 90% training data and 10% testing data. This split ensured that the model had a large, diverse training set while preserving a subset for unbiased performance evaluation on unseen data. The model was configured with 100 trees and a maximum of 10 layers, ensuring sufficient capacity to capture intricate patterns in the data. For more computational details refer to Section 3—Computational Details of the Supporting Information.

### 2.9 Model Training and Evaluation

During training, the RF classifier used the extracted features to learn patterns and relationships that distinguished between the different motion types. The *TreeBagger* function in MATLAB facilitated efficient training by constructing an ensemble of decision trees, each trained on a random subset of the data. Each tree is trained on a bootstrap sample of the training data, and the final prediction is made by aggregating the outputs (via majority vote) across all trees. A single decision tree operates by recursively splitting the feature space based on conditions of the form feature < threshold, selecting splits that maximize class separation using impurity metrics such as the Gini index. This hierarchical partitioning continues until stopping conditions (e.g., maximum tree depth or minimum leaf size) are met. The ensemble approach improves model robustness and reduces overfitting by averaging over multiple independently trained trees.^35,56^

The predictions from these trees were aggregated to produce the final classification for each motion segment. The baseline model performance achieved an accuracy of 78.6%, calculated as shown in eq 8. The confusion matrix for the baseline model is presented in Figure S3, demonstrating that DM is classified most accurately, followed by AD, ND, and CD.

8 $$Accuracy=\frac{Number\;of\;correct\;predictions}{Total\;number\;of\;predictions}\times 100$$

## 3 Results and Discussion

### 3.1 Feature Selection

We then identified 6 of the 14 original features that contributed most to distinguishing between different motion types in 3D trajectories (Figure 2). Three independent feature selection algorithms were applied: minimum redundancy maximum relevance (mRMR), neighborhood component analysis (NCA), and ReliefF (an extension of the Relief algorithm for multiclass feature selection). mRMR selects features that have maximum relevance to the target variable while minimizing redundancy among selected features.^57^ In practice, this means that mRMR identifies a set of features that provide meaningful information for the classification task, preventing the model from having redundant signals.^57,58^ NCA is a supervised learning method that optimizes feature weights to maximize classification accuracy, particularly within nearest-neighbor frameworks.^59^ By assigning higher weights to features that improve the classification of nearby data points, NCA highlights features that are most influential in distinguishing between different motion types. The ReliefF algorithm compares each data point with its nearest neighbors from both the same and different classes, quantifying how well each feature contributes to distinguishing data. ReliefF captures patterns in data, as it considers both local and global feature relevance.^60,61^

**Figure 2:**
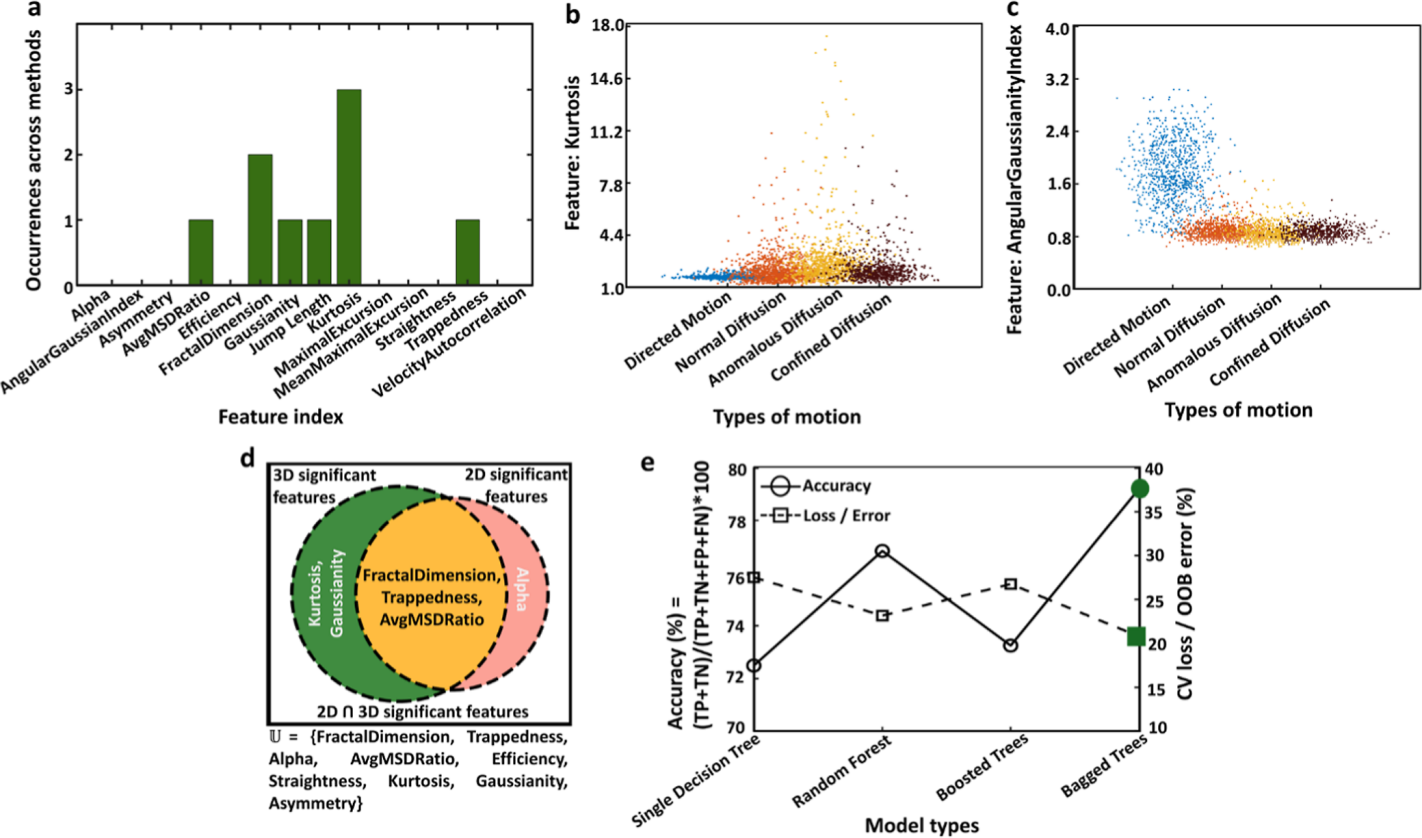
Enhanced model performance is achieved through feature selection. (a) Frequency of six features: kurtosis, fractal dimension, avg MSD ratio, Gaussianity, jump length, and trappedness, ranked in the top three by feature selection algorithms mRMR, NCA, and ReliefF. (b) Distribution of the critical feature Kurtosis across various motion types. (c) Distribution of the less critical feature, angular Gaussianity index. (d) Comparison of feature importance across 2D^41^ and 3D trajectories in the current study. (e) Comparison of model accuracy and cross-validation (CV) loss/out of the bag (OOB) error across single decision trees, RFs, boosted trees, and bagged trees, showing that bagged trees achieve the highest accuracy (79%) and the lowest CV loss (21%) with the 6-reduced feature model.

After applying these algorithms separately, we examined their respective feature rankings and selected the top three features from each method. This approach allowed us to build a final feature set composed of the most consistently high-ranking and informative features across all three methods. By leveraging the strengths of mRMR, NCA, and ReliefF individually, we ensured that the model’s feature set was diverse, highly relevant, and optimized for classifying complex 3D trajectories. The feature ranking from individual algorithms is shown in Figure S4. Kurtosis, fractal dimension, avg MSD ratio, Gaussianity, jump length, and trappedness were ranked as important by the three feature selection algorithms, as shown in Figure 2a. Kurtosis, measures the “tailedness” or extremity of the displacement distribution, providing information about the distribution’s shape and allowing for differentiation between motion types. For instance, DM tends to have lower kurtosis due to more predictable paths and AD or CD exhibit higher Kurtosis due to erratic or constrained trajectories.^39^ The distribution of Kurtosis values for all motion types is compared in Figure 2b. Fractal dimension measures the complexity of a trajectory and provides insights into the irregularity of the path, a key factor in differentiating between ND and constrained diffusion like AD or CD.^62^ Avg MSD Ratio assesses the relationship between MSD and time, offering critical information about overall movement patterns.^41^ Gaussianity evaluates how closely diffusion follows a Gaussian distribution, with deviations often signaling environmental heterogeneity or confinement.^63^ Jump length quantifies the magnitude of individual displacements, revealing whether movement is characterized by large steps or restricted displacements.^64^ Finally, trappedness evaluates the likelihood of a particle remaining confined, distinguishing between free and trapped diffusion.^65^ Together, these features characterize dynamics of 3D trajectories, allowing for effective classification across different diffusion behaviors.

Angular Gaussianity index,^66^ efficiency,^41^ and velocity autocorrelation consistently ranked lower in the feature selection algorithms. Angular Gaussianity index, for instance, focuses on angular displacement, which may not be as relevant for systems dominated by linear motion. In 3D, where both angular and linear movements are significant, this feature might not capture enough information to differentiate between trajectories. The distribution of angular Gaussianity indices for all motion types is compared in Figure 2c. Efficiency, which measures the directness of movement, might be overshadowed by more informative features like fractal dimension, which directly assesses path irregularities. Velocity autocorrelation, while important for understanding movement persistence, likely provides less insight compared to more direct measures of displacement, such as jump length and trappedness. Refer to Section 2—Trajectory feature extraction of the Supporting Information for a detailed explanation about the features.

Figure 2d highlights the similarities and differences in feature importance identified in our study of 3D classification compared to those presented in a recent 2D classification study.^41^ Here, we are focusing on the important features that are common in both the studies. Fractal dimension, trappedness, and avg. MSD ratio are critical features shared across both studies (orange region), signifying their consistency in characterizing diffusion dynamics irrespective of spatial dimensionality. These features address distinct aspects of particle motion: fractal dimension captures the complexity and irregularity of particle trajectories, trappedness quantifies confinement likelihood, and avg. MSD ratio correlates displacement and time to describe movement trends. Their shared importance between 2D and 3D classification suggests their broad applicability to diffusion classification.

In contrast, certain features emerge as uniquely significant to each study’s context. For instance, alpha, a scaling factor,^14^ was an important feature in the 2D study (pink region), but did not rank highly in our 3D comparison. In our 3D study, Kurtosis and Gaussianity stand out as critical features (green region). Kurtosis excels in identifying deviations from predictable motion through the tails of the displacement distribution, making it especially useful for distinguishing AD or CD.^67^ Similarly, Gaussianity assesses the conformity of particle displacement to a Gaussian distribution, capturing structural complexities or heterogeneity in 3D environments.^68^ These distinctions highlight the adaptation of feature selection to different spatial contexts and experimental needs.

We tuned and cross-validated the 6 high-ranking features using a single decision tree,^56^ RF,^69^ boosted trees,^70^ and bagged trees,^71^ and the results are shown in Figure 2e. We implemented k-fold cross validation,^72^ in which each data set is divided into k equal-sized subsets (folds). The model is trained on k-1 folds and tested on the remaining fold. This process is repeated k (*k* = 5, in this case) times, each time with a different fold used for testing. We computed the cross-validation (CV) loss^72,73^ for these models which is given by (eq 9)­

9 $$cross‐validation\;loss=\frac{1}{k}\sum_{i=1}^{k}Loss\left({ŷ}_{i},y_{i}\right)$$

where *k* is the number of folds in k-fold cross-validation (*k* = 5). ${ŷ}_{i}$ are the predicted values and *y*_*i*_ are the true values. The loss function $Loss\left({ŷ}_{i},y_{i}\right)$ quantifies the difference between the predicted values $\left({ŷ}_{i}\right)$ and true values (*y*_*i*_). For our classification, we included misclassification rate (1 – accuracy). We then averaged the loss across all *k* folds to provide an estimate of the model’s performance on unseen data. For the RF model, we measured the out-of-bag (OOB) error which is method for estimating the prediction error of a RF model.^74^ It leverages the bootstrap sampling technique used to train the individual trees in the forest. For each tree, about one-third of the data is not used for training (these data points are referred to as “out-of-bag” samples). OOB error is calculated by predicting the OOB samples using the tree that was not trained on them and then aggregating the results. The OOB error (eq 10) is computed as the average loss over all samples, where the loss function *L* is the misclassification rate.

10 $$OOB\;error=\frac{1}{N}\sum_{i=1}^{N}L\left({ŷ}_{i}^{OOB},y_{i}\right)$$

where *N* is the total number of samples, ${ŷ}_{i}^{OOB}$ is the aggregated prediction for the *i*-th sample from all trees that did not include this sample in their bootstrap sample and *y*_*i*_ is the true label of the *i*-th sample.

As shown in Figure 2e, the bagged tree (highlighted in green) model has the highest accuracy of 79% and least loss/error as 21%. The confusion matrix in Figure S5 shows that the model performs best on DM, followed by AD and ND, with the lowest performance on CD. The accuracy and loss/error for each model is given in Table S2. In comparison, the baseline model with all 14 features achieved an accuracy of 78%, while the tuned model (bagged tree), utilizing only 6 features identified through feature selection, slightly outperformed the baseline with an improved accuracy of 79%. These results show that feature selection and tuning make it possible to achieve better accuracy with fewer features and lower computational complexity.

#### 3.1.1 Feature Comparison between 3D and Projected 2D Trajectories

It is interesting to further compare the rankings of feature importance for 3D and 2D trajectories. We applied the same feature selection pipeline to two-dimensional (2D)-projected trajectories of our 3D data and found some consistencies between the 2D and 3D data as well as some differences. Representative 2D trajectories are provided in Figure S6, classification performance is summarized in Figure S7 and rankings of projected 2D features are shown in Figure S8 (compared with 3D feature ranking in Figure 2). Fractal dimension, Kurtosis, and jump length ranked among the most important features in both 3D and projected 2D models for our data, but with fractal dimension ranking first in 3D data and Kurtosis ranking first in 2D data. Trappedness was found to be important in both 3D and 2D, reflecting its ability to detect spatial confinement regardless of dimensionality. Wagner et al.^41^ similarly reported fractal dimension and trappedness as important parameters in 2D classification.

Gaussianity ranked more highly in the 3D analysis, suggesting that dimension reduction may reduce its discriminative power by smoothing out deviations from Gaussian step-size distributions. Velocity autocorrelation gained importance in projected 2D, where projection may enhance directional memory along observable axes. Avg. MSD ratio was consistently ranked as important in 2D and 3D. These findings can also be considered in the context of prior work by Kowalek et al.,^43^ who applied both feature-based and deep learning approaches to classify diffusion modes in 2D single-particle tracking data. Their study identified MSD ratio and diffusion constant, which can be equated to velocity autocorrelation in our analysis, as similarly important features for distinguishing simulated motion types.

We further compared feature relevance to distinct motion-types across 3D and projected 2D models. We trained one-vs-rest classifiers for each motion type, DM, ND, AD, and CD and computed the change in out-of-bag error (ΔOOB_*f*_) when each feature was permuted. This quantifies the importance of each feature *f* for correctly classifying a particular class. The results, normalized between 0 and 1 per class, are visualized as heatmaps in Figure S9. In 3D, Figure S9a, fractal dimension is the most important feature for all classes except CD, with an importance score of 1.00 for DM, ND, and AD, and a still substantial 0.40 for CD. This result highlights the role of fractal geometry in distinguishing motion types in 3D.

For CD, trappedness dominates (1.00), which is important for capturing spatial confinement in 2D and 3D. Trappedness is also highly relevant for AD (0.63), suggesting partial confinement or heterogeneous environments in these trajectories. In projected 2D Figure S9b, the feature landscape shifts after dimensional reduction. Fractal Dimension remains the most discriminative feature for DM, ND, and AD (all 1.00), but its importance for CD drops to 0.53. Kurtosis becomes especially prominent for DM (0.81), likely due to increased asymmetry in projected displacement distributions. Jump length is the second most important feature for ND (0.62) and moderately important for CD (0.27). For AD and CD, Trappedness is the key feature (1.00 for both), indicating robust confinement detection even in 2D. Other features, such as AvgMSDRatio and Velocity Autocorrelation, contribute modestly across classes (maximum 0.53 for CD in avg MSD ratio and 0.42 for ND in velocity autocorrelation).

These findings suggest that spatial dimensionality can influence feature discriminability, and our work complements previous studies^41,43^ by emphasizing the value of spatial context in diffusion mode classification.

### 3.2 Hyperparameter Optimization

Bayesian optimization^45,46^ and random search^45^ were compared to further improve the classification by identifying the best set of nonlearnable hyperparameters governing the training process and structure while also improving accuracy, computational efficiency, and generalizability (Figure 3).^45^ The two hyperparameters used were learning cycles and minimum leaf size. The number of learning cycles determines the number of iterations during training and influences the depth of learning. Minimum leaf size defines the smallest number of samples allowed in a decision tree leaf, balancing model complexity and overfitting. These hyperparameters were critical to the performance of the bagged ensemble model (*fitcensemble*) used in this study, which implements bootstrap aggregating (*bagging*) to combine predictions from multiple decision trees, reducing variance and improving classification performance.

**Figure 3:**
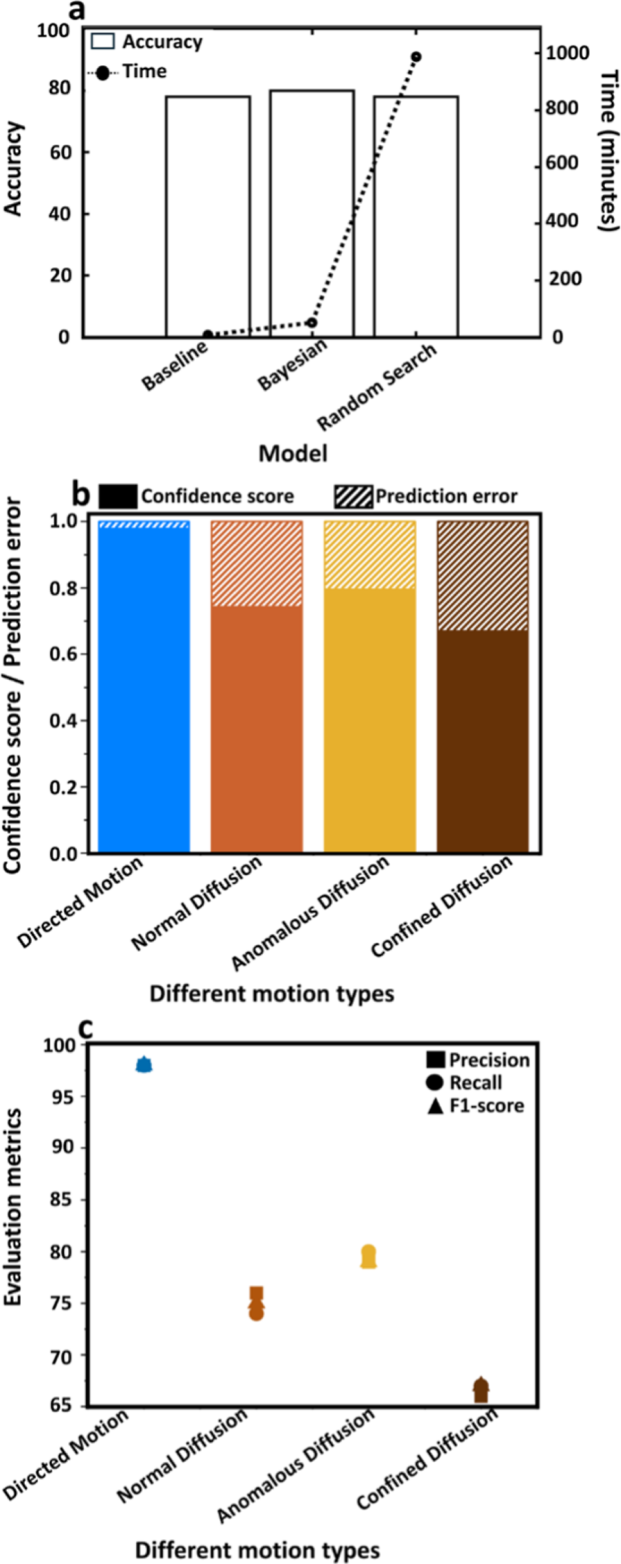
Hyperparameter optimization. (a) Comparison of accuracy and computational time of Bayesian optimization, and random search with the baseline model. (b) A bar chart displays the model’s prediction errors and corresponding confidence scores over four motion classifications: DM, ND, AD, and CD. (c) Precision, recall, and *F*1-scores for each type of motion, underscoring the model’s enhanced ability to correctly identify DM, followed by ND, AD, and CD, in that order.

Figure 3a compares the optimization techniques with a baseline model in terms of accuracy and computational time. Bayesian optimization uses a probabilistic approach to efficiently explore the hyperparameter space, focusing on the most promising regions to minimize cross-validated loss. Using MATLAB’s *bayesopt* function, we defined a custom objective function that calculated the average cross-validated loss with 5-fold cross-validation on the training data. The parameter ranges were set between 150–500 for the number of learning cycles and 20–50 for the minimum leaf size. Bayesian optimization achieved the optimal configuration of 499 learning cycles and 20 minimum leaf size, resulting in the highest accuracy of 80% in just 100 min. The confusion matrix is shown in Figure S10a. While the confusion matrices for the baseline and optimized models appear broadly similar, the optimized model achieves higher overall classification accuracy by reducing misclassification rates, particularly for challenging cases involving ND, AD, and CD. For instance, the percentage of ND samples misclassified as CD decreases from 20% to 18% in the optimized model. Similarly, CD samples misclassified as ND decrease from 24% to 18%. The proportion of AD samples misclassified as CD is reduced from 18% to 15%. Across all classes, the overall correct classification rate improves, demonstrating that hyperparameter tuning enhances the model’s ability to distinguish between motion types. These quantitative gains underscore the value of model optimization for achieving robust and reliable single-particle motion classification and motivate the need for better tuning models for future improvement.

To establish that boundaries were chosen appropriately, we performed Bayesian optimization on a subset of 50,000 trajectories randomly selected from the training set while preserving class distribution and trajectory variability. The convergence plot (Figure S10b) shows that the cross-validated classification loss steadily decreased during early iterations and plateaued after approximately 30 rounds, indicating stable convergence. The resulting hyperparameter configuration generalized well to the full training and test sets, achieving a final test accuracy of 80%, consistent with the optimized loss observed on the subset. While the optimal hyperparameters were near the edge of the search space, the convergence analysis suggests that they lie within the limitation of the chosen bounds.

Here it should be noted that early stopping is typically used in sequential learners such as boosting frameworks, where models can be evaluated incrementally during training. However, our model employs bagging, where trees are trained independently and in parallel, making early stopping impractical. We therefore relied on Bayesian optimization over a defined hyperparameter space, using cross-validated classification loss as the objective. This is a standard and appropriate method for tuning bagged ensembles (e.g., fitcensemble with “Bag”),^71^ ensuring optimal configuration without relying on sequential evaluation.

In contrast, Random search explores the hyperparameter space by randomly selecting combinations within predefined ranges. While straightforward, it is less efficient as it does not leverage prior evaluations to guide the search process. Random search uses MATLAB’s *randsample* function, randomly sampling 100 combinations of the same parameter ranges. The best configuration identified by Random search was 464 learning cycles and 20 minimum leaf size, with an accuracy of 78%. Most importantly, this method required over 1000 min to complete, confirming the higher efficiency of Bayesian optimization.

The impact of these optimization techniques, along with their comparison to the baseline model, is summarized in Figure 3a. While the baseline model achieved similar accuracy to Random search, it did so without tuning and required significantly less computational time. In contrast, Bayesian optimization outperformed both the baseline and Random search, achieving the highest accuracy with relatively low computational demands. (Refer to Section 3—Computational Details of the Supporting Information). After optimization, the bagged ensemble models were trained and evaluated using the identified hyperparameter configurations. For both techniques, we used MATLAB’s *fitcensemble* function to train the model, with the decision trees defined using the templateTree function to incorporate the optimized minimum leaf size. The final models were tested on a holdout data set, predictions were generated, and performance was evaluated.

Additional comparisons that support the superiority of Bayesian analysis are shown in Figure 3b,c. Figure 3b presents a bar chart showing confidence scores and prediction errors for four motion types: DM, ND, AD, and CD. In general, CS and PE are defined as (eqs 11 and 12)­

11 $${confidence\;score}_{i}=\frac{{correctly\;classified\;samples}_{i}}{{total\;samples}_{i}}$$

12 $$prediction\;error_{i}=1-confidence\;score_{i}$$

Correctly classified samples_*i*_ is the number of correctly classified samples for class *i* which is given by the diagonal element of the confusion matrix for that class. Total samples_*i*_ is the total number of samples for class *i* which is given by the sum of all elements in the row corresponding to class *i*. DM achieved the highest confidence score (0.98) and the lowest prediction error (0.02).

Figure 3c provides a detailed breakdown of precision, recall, and *F*1-scores for each motion type, showcasing the model’s classification performance. For DM, the precision, recall, and *F*1-score are all 0.98, highlighting exceptional performance in identifying this motion type. For ND, the precision is 0.76, recall is 0.74, and *F*1-score is 0.75, reflecting slightly lower but consistent performance. For AD, the precision is 0.79, recall is 0.80, and *F*1-score is 0.79, indicating balanced predictions. Finally, for CD, the precision is 0.66, recall is 0.67, and *F*1-score is 0.67, showing this motion type as the most challenging for the model to classify. These results rank the motion types in terms of performance as DM (best), followed by ND, AD, and CD.

The analysis in Figure 3c highlights the performance of the model across different motion types. The nearly identical precision (0.98), recall (0.98), and *F*1-score (0.98) for DM indicate that the model is equally effective at correctly identifying true positives and minimizing false negatives. Similarly, the close alignment of metrics for other motion types, such as AD, suggests the model does not overly favor precision (correctness of positive predictions) at the expense of recall (ability to identify all actual positives), or vice versa. When precision and recall are similar, it indicates that the model is well-calibrated, meaning it neither overpredicts nor under-predicts any specific class. The *F*1-score, a harmonic mean of precision and recall, remains consistent with these values, further validating the model’s reliable and balanced performance. The uniformity across metrics also demonstrates that the model avoids significant biases toward specific types of errors, ensuring strong classification for all motion types. Moreover, the similarity in precision, recall, and *F*1-scores across motion types indicates minimal trade-offs between identifying as many relevant instances as possible (high recall) and ensuring the correctness of predictions made (high precision). This balance is critical in achieving a robust and reliable classification model, particularly for tasks involving multiple distinct classes.^75^

### 3.3 Simulated Experimental Data Analysis

Figure 4 presents the workflow for classification of different types of motion in simulated microscopy movies that mimic experimental conditions using our optimized model (simulation details provided in Section 4—Movie Simulations in Supporting Information). These simulated movies include all four types of motion, DM, ND, AD, and CD, as well as movies with combinations of two motion types, reflecting scenarios more commonly observed in biological systems (Figure S11). The trajectories from the simulated movies were extracted using the KNOT (knowing nothing outside tracking) particle tracking algorithm (Details in Section 4.2—Recovering Trajectory Data from Simulated Movies in Supporting Information).^76–82^

**Figure 4:**
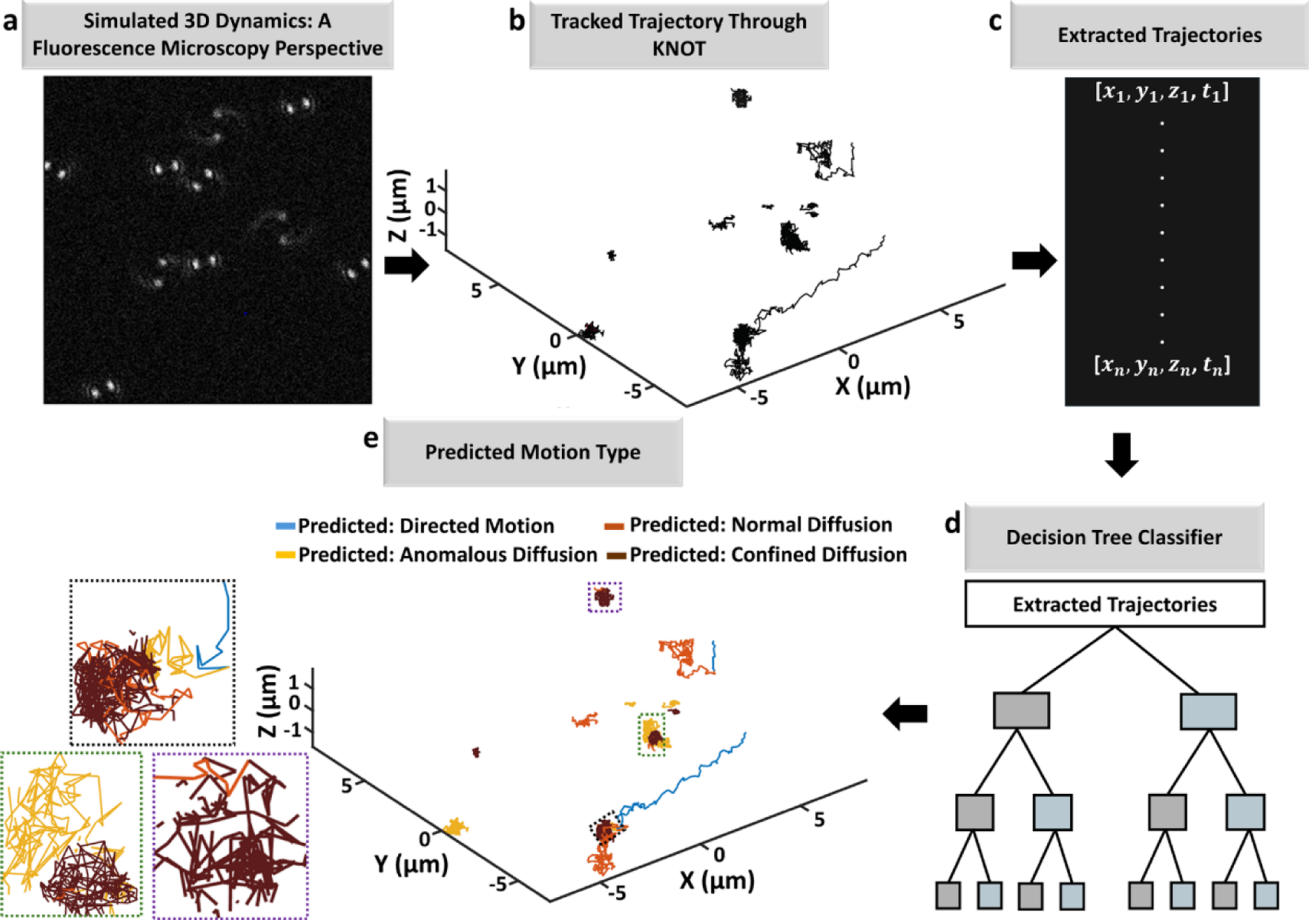
Workflow for classification of different types of motion in simulated movies mimicking experimental conditions using the optimized model (a) microscopy movies are simulated to mimic experimental conditions. (b) Particle trajectories are extracted from the movies using the KNOT particle tracking algorithm,^76^ producing time-sequenced spatial coordinates [*x*,*y*,*z*] over time *t*. (c) Trajectories are processed through calibration and filtering procedures after which features are calculated to characterize the motion dynamics. (d) The optimized model developed using a decision tree classifier is applied to classify motion types such as DM, ND, AD, or CD based on learned features. (e) The classified motion types are visualized in 3D, with trajectories color-coded by their predicted motion type, demonstrating the classifier’s ability to analyze complex motion dynamics in simulated data. This workflow validates the application of the model to conditions resembling real experimental scenarios.

The workflow starts with generating 3D fluorescence microscopy movies to replicate experimental imaging conditions, capturing the dynamic behavior of particles as shown in Figure 4a. These simulated movies are then analyzed using the KNOT tracking algorithm^76^ to extract 3D trajectories, producing a time series of spatial coordinates [*x*,*y*,*z*] over time *t*, as shown in Figure 4b. In Figure 4c after trajectories undergo calibration and filtering, motion-related features are extracted to characterize their dynamic behavior. To ensure data quality, the code applies trajectory filtering and segmentation—removing short or disconnected trajectories and applying threshold-based criteria to retain, analyzable data (Detailed in Section 5—Extracted Trajectory Data Analysis of Supporting Information). Motion features are calculated over sliding time windows to capture dynamic behavior at finer temporal resolution, enabling the model to detect subtle transitions in motion.

These features are then input into the optimized decision tree classifier, as illustrated in Figure 4d, which is trained to distinguish between four motion types: DM, ND, AD, CD. The classifier evaluates the features and assigns each trajectory segment into one of these categories. Finally, the classified trajectories are visualized in 3D (Figure 4e), with each motion type represented by a distinct color, providing an intuitive understanding of the classifier’s performance. By integrating calibration, trajectory matching, feature extraction, and classification, this workflow ensures a pipeline for classification of 3D single particle motion, demonstrating the applicability of our optimized model for both simulated and experimental data sets.

Unfortunately, the number of trajectories extracted from the simulated movies is insufficient to reliably estimate the true accuracy of the predicted classification. To assess the effects of PSF (point spread function) blur, camera noise, and tracking errors, we applied our model to both extracted and original simulated trajectories and compared the results. The classification accuracies observed for trajectories extracted from the movies of 17 individual particles were: DM (64%), ND (61%), AD (78.), and CD (47%). For the original simulated trajectories, the accuracies were: DM (64%), ND (73%), AD (77%), and CD (62%). These results show that the model performs consistently for DM and AD between simulated and tracked data. The reduced accuracy for ND and CD stems from the added difficulty of recovering these motion types from noisy, PSF-based movies. It is important to note that the results obtained in this part of the study differ from the previously reported 80% accuracy due to the use of a limited data set. Expanding the data set in future studies should help improve model performance and generalizability.

While dynamic localization error (i.e., motion blur) is not included in our simulations, static localization uncertainty (i.e., PSF convolution, photon noise, and KNOT tracking artifacts) is implicitly incorporated through our image-based simulation and tracking pipeline. By generating 3D microscopy movies using scalar diffraction theory and realistic PSFs, followed by KNOT tracking, we introduce spatial uncertainty and fragmentation that closely mimic real experimental conditions. Overall, this comparison highlights the challenges of classifying trajectories from realistic movies. In future work, we plan to improve tracking algorithms, enhance noise modeling, and expand simulated movie data sets to improve performance, particularly for more challenging motion types such as CD.

### 3.4 Experimental Validation of ND

To test the model’s applicability to real experimental data, we applied it to 3D single-particle trajectories of fluorescent beads diffusing freely in 40% glycerol, a well-characterized system expected to follow ND (Detailed in Section 6—Experimental Validation of ND). Using the same imaging and tracking pipeline described in Section 5—Extracted Trajectory Data Analysis of Supporting Information we recorded trajectories and applied our trained classifier. The model classified 86% of trajectories as ND, with smaller fractions identified as DM (6%), DM + ND (3%), and ND + CD (5%), and no trajectories as purely AD or CD (Figure S12). These minority classifications likely reflect brief surface interactions, drift, or noise, and offer the possibility, with expanded training and hyperparameter tuning, to understand rare events in transport data. Overall, these results confirm that our simulation-trained classifier generalizes effectively to real experiments and reliably detects ND, providing initial validation of our framework under experimental conditions.

### 3.5 Application to Experimental Data (Polymer Brushes)

To assess the applicability of our model in experimental systems, we reanalyzed previously published experimental data from tracking 8000 trajectories of anionic dye molecules diffusing within a cationic poly­(2-(*N*,*N*-dimethylamino)­ethyl acrylate) (PDMAEA) polymer brush at pH 3.^47^ The dynamics of solutes within polymer brushes are complex due to the interplay of electrostatic interactions, steric confinement, spatial heterogeneity, and chain architecture. Densely grafted polyelectrolyte brushes such as PDMAEA create a structurally anisotropic and dynamic medium in which solutes can exhibit a wide range of motion behaviors that are difficult to resolve.^47,83^ This complexity necessitates advanced tools, such as our optimized ML model, to identify and classify MM types in 3D SPT. In the original work, the authors broadly categorized probe motion into “confined” and “unconfined” groups based on qualitative radius of gyration analysis.^47^ In this study, we apply our model to the same data set to provide a more detailed analysis of the observed dynamics.

As shown in Figure 5a, the experimental system consists of a cationic PDMAEA polymer brush grafted to a surface and imaged using 3D SPT fluorescence microscopy. The brush is composed of positively charged polymer chains extending vertically from the surface, forming a dense, hydrated layer. Alexa Fluor 546, an anionic dye, is introduced as the probe molecule. Due to its negative charge, the probe interacts electrostatically with the brush and experiences local variations in polymer density, leading to complex transport behavior.

**Figure 5:**
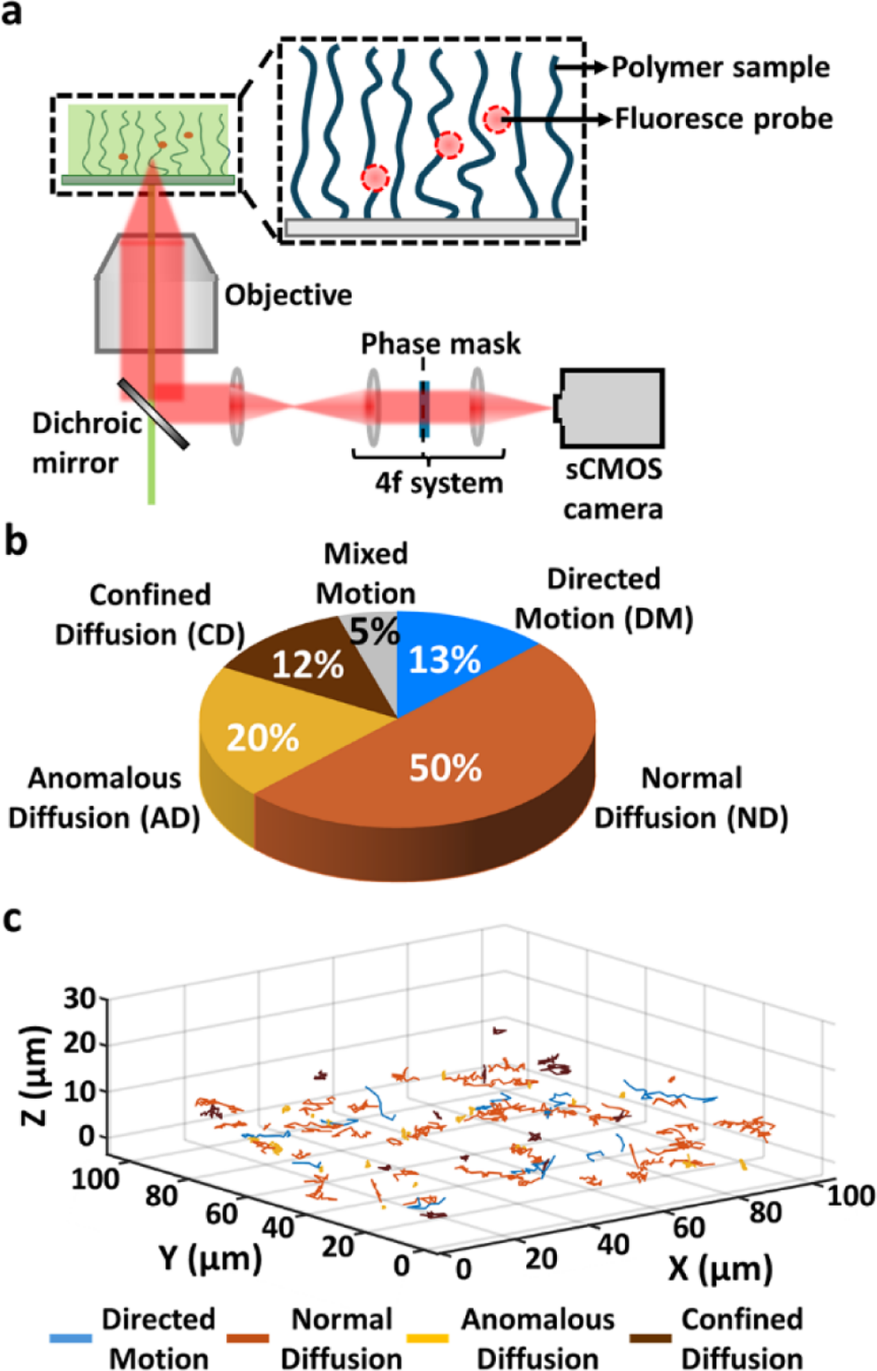
The optimized model is used to analyze the different types of motion for anionic dyes in a cationic polymer brush. (a) A cartoon representation of a cationic poly­(2-(*N*,*N*-dimethylamino)­ethyl acrylate) (PDMAEA) brush with Alexa Fluor 546, an anionic dye, used as the probe. (b) The distribution of motion types in the polymer brush. (c) A 3D visualization of random trajectories across all predicted motion types generated by the optimized model.

Applying our model to this data set enabled a more refined classification of probe dynamics within the PDMAEA brush compared with that in the original analysis (Figure 5b,c). These results provide new insight beyond the binary classification offered in Fan et al.*‘s* study,^47^ which grouped 77% of trajectories as “unconfined” and 23% as “confined” based on radius of gyration threshold. As illustrated in Figure 5b, most trajectories are identified as ND, comprising approximately 50% of the data set. This is consistent with the “unconfined” motion identified in the prior work and with the notion that these molecules undergo unhindered spatial exploration. About 20% of the trajectories are classified as AD, reflecting probe dynamics that deviate from classical diffusion, likely due to local confinement, spatial crowding, or local heterogeneities. DM accounts for 13% of trajectories, while CD is identified in 12% of the trajectories, representing molecules that remain trapped within localized subdomains, either between chains, near the grafting surface, or in dense polymer regions. Figure 5c presents a 3D visualization of randomly selected probe trajectories, color-coded by their predicted motion type.

Interestingly, 5% of the trajectories exhibit MM behaviors, transitioning between two or more diffusion modes during the observation period, indicative of spatially heterogeneous environments or dynamic interactions with the brush matrix. Figure S13 further quantifies the distribution of MM trajectories in which a probe transitions between multiple dynamic modes. This histogram underscores that while most probes exhibit a dominant motion type, a subset undergoes mode-switching during their trajectory. Such behavior is likely driven by spatial variations in brush density, local charge distribution, or transient interactions, further reinforcing the value of a segment-wise classification approach.

The reanalysis reveals that among the trajectories previously labeled as “unconfined” in the original study,^47^ our model reveals that 73% are best described as either ND (58%) or DM (15%). This updated breakdown closely mirrors the 77% “unconfined” fraction originally reported, suggesting that these probes move with relatively fewer constraints. The DM component likely reflects probe movement steered by localized electrostatic interactions with the charged brush environment rather than directional alignment of the polymer chains. In contrast, within the “confined” population previously described, 23% of trajectories are now reassigned as AD (51%) or ND (25%), suggesting that their behavior arises from complex local interactions rather than simple physical trapping. These analyses are quantitatively summarized in Figure S14. This refined classification highlights the ability of ML to reveal subtle differences in transport dynamics, offering insight into the heterogeneous and dynamic nature of the polymer brush environment.

## 4 Conclusions

This study demonstrates the efficacy of ML in classifying different types of motion in 3D single-particle trajectories. By implementing decision tree algorithm with a rigorous feature selection process, we identified six critical features, kurtosis, fractal dimension, average MSD ratio, Gaussianity, jump length, and trappedness, that are highly relevant for 3D motion classification. Hyperparameter optimization, particularly through Bayesian optimization, proved to be the most effective method for tuning model parameters, achieving a balance between high accuracy (80%) and low computational time. Application to an experimental sample highlighted the potential of ML-driven classification to provide insights into heterogeneous 3D transport dynamics in complect materials and biological samples. To assess the role of spatial dimensionality, we compared classification performance and feature importance in 3D trajectories versus their projected 2D counterparts. Beyond global feature importance, our class-specific permutation importance analysis highlighted how different motion types rely on distinct features. Future work will focus on further refining the ML models using methods such as deep learning algorithms to improve classification accuracy, particularly for challenging motion types like CD, where the current model showed relatively lower performance.

## Supplementary Material



## Data Availability

All code developed for this study is openly available on GitHub at [https://github.com/LandesLinkLab/3D-SPT-Classification]. The repository includes scripts for simulations, trajectory feature extraction, and data analysis. Any additional data sets generated and analyzed during the current study are available from the corresponding author upon reasonable request.

## Notes

Detailed content on the simulation of motion models; trajectory feature extraction; computational details; movie simulations; extracted trajectory data analysis; experimental validation of ND; supporting figures; supporting tables; and references (PDF)
