# Supplementary material for: Feature Selection and Hyperparameter Optimization for Machine Learned Classification of 3D Single-Particle Tracking
Source: Chem Biomed Imaging. 2025 Aug 14;4(1):79–91. doi: 10.1021/cbmi.5c00057 (PMC12848716; doi:10.1021/cbmi.5c00057)
Supplement: Supplementary file 1 [file im5c00057_si_001.pdf]

SUPPORTING INFORMATION

Feature Selection and Hyperparameter Optimization for Machine Learned Classification of 3D Single-Particle Tracking

Jagriti Chatterjee,[1,2] Subhojyoti Chatterjee,[1,2] Emil Gillett,[1,2] Nikita Kovalenko,[1,2] Dongyu Fan,[3,4] Christy F. Landes*[1,2,3,4,5,6]

[1]Department of Chemistry, University of Illinois at Urbana-Champaign, Urbana, IL 61801, United States

[2]Department of Chemistry, Rice University, Houston, TX 77005, United States

[3]Department of Chemical and Biomolecular Engineering, University of Illinois at Urbana-Champaign, Urbana, IL 61801, United States

[4]Department of Chemical and Biomolecular Engineering, Rice University, Houston, TX 77005, United States

[5]Department of Electrical and Computer Engineering, University of Illinois at Urbana-Champaign, Urbana, IL 61801, United States

[6]Department of Electrical and Computer Engineering, Rice University, Houston, TX 77005, United States

*Corresponding Author: cflandes@illinois.edu

TABLE OF CONTENTS

Topics	Page Number
**Simulation of Motion Models**	3
**Trajectory Feature Extraction**	4
**Computational Details**	11
**Movie Simulations**	11
**Extracted Trajectory Data Analysis**	14
**Experimental Validation of Normal Diffusion**	16
**Supporting Figures**	18
**Supporting Tables**	32
**References**	34

1. Simulation of Motion Models

In this section, we give the general definitions and motion models used for simulating 3D trajectories. We simulate 4 basic types of motion: directed motion (DM), normal diffusion (ND), anomalous diffusion (AD) and confined diffusion (CD). In general, the analysis of these trajectories is done by mean square displacement (MSD) method, which is defined as **(Eq. S1)**:

$$\langle r^2(n)\rangle = \frac{1}{N-n}\sum_{k=1}^{N-n}|x_{k+n} - x_k|^2 , \qquad n = 1, \dots\dots, N-1 \tag{S1}$$

where, N represents the number of consecutive two-dimensional positions of a particle, denoted as $x_k = (x_k, y_k)$, recorded at regular time intervals $\boldsymbol{\Delta t}$ over a total duration $T = (N-1)\boldsymbol{\Delta t}$. A step is defined as movement from x_k to x_{k+n} , with the step length defined by the Euclidean distance $|x_k - x_{k+1}|$. If only a segment of the trajectory x_k to x_{k+l} is considered, it is referred to as a sub-trajectory.[1]

Defined by Saxton, the MSD function of the 4 basic types of motion in 3D are represented by:

For DM (Eq. S2):

$$\langle r^2(n)\rangle = 6Dn\Delta t + (vn\Delta t)^2 \tag{S2}$$

Where, v is the velocity of DM

For ND (Eq. S3):

$$\langle r^2(n)\rangle = 6Dn\Delta t \tag{S3}$$

For AD (Eq. S4):

$$\langle r^2(n)\rangle = 6D(n\Delta t)^{\alpha} \tag{S4}$$

Where, α is the anomalous exponent

For CD (Eq.S5):

$$\langle r^2(n)\rangle \simeq r_c{}^2[1 - C_1 \exp(-6C_2 Dn\Delta t/r_c{}^2] \quad \text{(S5)}$$

Where, r_c is the radius of confinement and C_1 and C_2 are the characteristics of shape confinement.

2. Trajectory Feature Extraction

Adopting the methodology proposed by Wagner et al., we used their nine features namely: Alpha, Asymmetry, Efficiency, Fractal Dimension, Gaussianity, Kurtosis, Mean squared displacement ratio, Straightness, Trappedness.[1] In this current work, we have also incorporated features like Angular gaussianity index, Velocity autocorrelation, Maximal excursion, Mean maximal excursion and Jump length. [2] Below are the explanations of each features that were incorporated in this work:

a. **Alpha:** This feature is the representation of the scaling exponent derived from a power-law fit of the MSD given in Eq.S1. It is then calculated from the slope of the log-log plot of MSD versus time lag as **(Eq.S6)**:

$$\alpha = \frac{d\log\langle r^2\rangle}{d\log\langle n\rangle} \quad \text{(S6)}$$

and helps to classify the different types of diffusion. When $\alpha \approx 1$, we get ND, for DM $\alpha > 1$, for AD and CD $\alpha < 1$.

b. **Angular gaussianity index:** This feature is used understand the quantification of angular changes in a single particle trajectory from a Gaussian distribution. It helps to understand the non-Gaussian behavior in the angular displacement of a particle that has constrained

motion in a medium. For a given 3D trajectory with positions (x, y, z) over time, we first computed the displacement vectors between two consecutive points **(Eq.S7)**:

$$\Delta x_i = x_{i+1} - x_i \text{ , } \Delta y = y_{i+1} - y_i \text{ , } \Delta z = z_{i+1} - z_i \tag{S7}$$

Then the magnitude of each displacement vector is calculated by **(Eq.S8)**:

$$v_{norm,i} = \sqrt{(\Delta x_i)^2 + (\Delta y_i)^2 + (\Delta z_i)^2} \tag{S8}$$

We then next determine the main vector (m) of the trajectory from the start to the end **(Eq. S9)** and then compute the magnitude of the main vector **(Eq.S10)**

$$m = [x_{end} - x_{start}, y_{end} - y_{start}, z_{end} - z_{start}] \tag{S9}$$

$$m_{norm} = \|m\| = \sqrt{(x_{end} - x_{start})^2 + (y_{end} - y_{start})^2 + (z_{end} - z_{start})^2} \tag{S10}$$

Further we calculate the angular deviations by computing angle (θ_i) between the min vector and each displacement vector using the dot product formula **(Eq.S11)**:

$$\theta_i = \arccos\left(\frac{m.[\Delta x_i, \Delta y_i, \Delta z_i]}{m_{norm} . v_{norm}, i}\right) \tag{S11}$$

where, $m.[\Delta x_i, \Delta y_i, \Delta z_i]$ is the dot product of the main vector and the displacement vector. After calculating the differences between consecutive angular deviations **(Eq. S12)**, we create a histogram, of the angular changes over a number of bins (-π to π) as in **Eq. S13,**

$$d\theta_i = \theta_{i+1} - \theta_i \tag{S12}$$

$$counts = histcounts\,(d\theta, edges) \tag{S13}$$

We also compute the standard deviation (σ) of the histogram counts to quantify how much the distribution deviates from a Gaussian distribution by **(Eq. S14)**:

$$\sigma = std\,(counts) \tag{S14}$$

Finally, we compute the Higher-Order Gaussianity Index using **(Eq.S15)**:

$$HGI = \frac{1}{n^2\left(\frac{1}{n}-1\right)}\sum_{i=1}^{n}\left(\frac{counts(i)}{n}-\frac{1}{n}\right)^4 \tag{S15}$$

where:

n is the number of bins in the histogram, and, $counts(i)$ represents the number of occurrences in the i -th bin.[3]

c. **Asymmetry:** As defined by Saxton[4], the asymmetry a of the particle's path is examined by the eigenvalues of the radius of gyration tensor. High asymmetry indicates anisotropic motion , which could be either DM or CD **(Eq.S16)**.

$$a = -\log\left(1-\frac{(\lambda_1-\lambda_2)^2}{2\,(\lambda_1+\lambda_2)^2}\right) \tag{S16}$$

where, λ_1 and λ_2 are the eigenvalues of the radius of gyration tensor T_{ij}, which is given by **(Eq.S17)**:

$$T_{ij} = \frac{1}{N}\sum_{k=1}^{N}(x_{k,i}-\bar{x}_i)(x_{k,j}-\bar{x}_j) \tag{S17}$$

T_{ij} is the element of the tensor at row i and column j, N is the total number of points in the trajectory, $x_{k,i}$ is the i-th coordinate of the k-th point in the trajectory, $\bar{x}_i$ is the mean of all points along i-th dimension. Lower value indicates that the angular changes of the particle's trajectory are close to Gaussian distribution, suggesting the occurrence of ND, while higher value indicate significant deviations from the Gaussian distribution , implying either DM or confinement properties in a trajectory.

d. **Average MSD Ratio:** This feature compares the MSD values at different time lags, that is helpful in detection of particle's confinement or DM (Eq. 13). The ratio approaches zero as the the time lag increases, indicating the that for ND, the ratio is 0, for AD and CD >0 and for DM < 0 **(Eq.S18)**.

$$\langle r^2 \rangle_{n_1,n_2} = \frac{\langle r_{n_1}^2 \rangle}{r_{n_2}^2} - \frac{n_1}{n_2} \tag{S18}$$

e. **Efficiency:** Efficiency implies the effectiveness of a particle movement from its starting point to its end point, thus measuring the linearity of a trajectory, helping to detect DM **(Eq. S19)**

$$E = \frac{|x_{N-1} - x_0|^2}{(N-1)\sum_{k=1}^{N-1} |x_k - x_{k-1}|^2} \tag{S19}$$

f. **Fractal Dimension:** As defined by Katz and George, the fractal dimension quantifies the complexity and space-filling nature of the trajectory **(Eq.S20)**. [5]

$$D_f = \frac{\log N}{(NdL^{-1})} \tag{S20}$$

where, L is the total length of the path, N is the number of steps and d is the largest distance between any two positions. A higher value of fractal dimension indicates more convolution, while lower value suggests directionality in the movement. Thus, for DM the value is around 1, for ND the value is around 2 and for AD and CD will have values around 4.

g. **Gaussianity:** According to Ernst et al., the Gaussianity feature of a trajectory helps to understand the anomalous diffusion modes, which is given by **(Eq.S21)**:

$$g(n) = \frac{\langle r_n^4 \rangle}{2\langle r_n^2 \rangle^2} \tag{S21}$$

where, $\langle r_n^4 \rangle$ is the quatric moment of a trajectory given by **(Eq.S22)**:

$$\langle r_n^4 \rangle = \frac{1}{N-n}\sum_{k=1}^{N-n} |X_{k+n} - X_i|^4 \tag{S22}$$

The gaussianity value close to zero indicates ND , while deviations suggests other dynamics like DM and CD.[6]

h. **Jump Length :** The distance a particle travels between consecutive time step is referred to as jump length. ND is supposed to have broad distribution of jump lengths, DM will exhibit consistent jump length, while AD and CD will have limited jump length characterizing their confinement property. [7]

The formula to compute the jump length J_i between positions at consecutive time steps i and $i+1$ in a 3-dimensional space is **(Eq.S23)**:

$$J_i = \sqrt{(x_{i+1} - x_i)^2 + (y_{i+1} - y_i)^2 + (z_{i+1} - z_i)^2} \tag{S23}$$

where:

(x_i, y_i, z_i) is the position of the particle at time step i

$(x_{i+1}, y_{i+1}, z_{i+1})$ is the position of the particle at the next time step $i+1$

i. **Kurtosis:** This feature provides information about asymmetry and peakedness of the distributed points in a trajectory. Kurtosis (K) is computed by projecting the positions onto the dominant eigenvector of the radius of gyration tensor, allowing for the assessment of the trajectory's shape and how sharply it is distributed around its central axis **(Eq.S24)**:

$$K = \frac{1}{N}\sum_{k=1}^{N} \frac{\left(x_i^p - x^{\bar{p}}\right)^4}{\sigma_{x^p}^4} \tag{S24}$$

where, x^p represents one dimensional projected positions, the mean projected position is given by $x^{\bar{p}}$ and σ_{x^p} is the standard deviation of x^p.[8]

j. **Maximal excursion:** This feature is defined by the maximum distance a particle has traveled from its initial point, normalized by the Euclidean distance between the first and last points. The equation is given by **(Eq.S25)**:

$$ME = \frac{\max(|X_{k+1} - X_k|)}{X_N - X_0} \tag{S25}$$

k. **Mean Maximal excursion:** The mean maximal excursion can serve as an alternative method to the mean squared displacement (MSD) for determining the anomalous diffusion exponent. This is defined as the normalized maximum distance traveled by a particle, **(Eq.S26)**

$$T_n = \frac{\max(|X_i - X_0|)}{\hat{\sigma}_N^2 (t_N - t_0)} \tag{S26}$$

where, $\hat{\sigma}_N$ is the standard deviation estimator given by **(Eq.S27)**:

$$\hat{\sigma}_N^2 = \frac{1}{2N\Delta t} \sum_{l=1}^{N} \|X_l - X_{l-1}\|_2^2 \tag{S27}$$

l. **Straightness:** This feature measures how straight a trajectory is, thus providing a way to distinguish between DM and other motion types. The straightness *S* of a trajectory is given by the total displacement to the sum of step lengths **(Eq.S28)**:

$$S = \frac{|x_{N-1} - x_0|}{\sum_{i=1}^{N=1} |x_i - x_{i-1}|} \tag{S28}$$

m. **Trappedness:** Trappedness feature computes the probability that a diffusing particle is confined or "trapped" within a bounded region over a period of time. This feature helps to understand the particle behaviors in environments having physical barriers or constraints. As defined by Saxton, the probability P_t that a particle is trapped within a boundary is given by **(Eq.S29)**:

$$P_t = 1 - \exp\left(0.731 - 9.557\,\frac{D.t}{r_0^2}\right) \tag{S29}$$

where:

P_t is the probability that the particle is trapped within a region of radius, r_0 during time t and D is the short-time diffusion coefficient, which represents how fast the particle diffuses at short timescales. Here, r_0 is dynamically computed for each trajectory window as half of the maximum distance between any two points in the trajectory. Specifically, we identify the largest separation between any two positions within the trajectory segment, and set r_0 as given by **(Eq.S30)**:

$$r_0 = 0.5 \times \max\left\{\sqrt{\left(x_i - x_j\right)^2 + \left(y_i - y_j\right)^2 + \left(z_i - z_j\right)^2}\right\} \tag{S30}$$

where the maximum is taken over all point pairs (i, j) in the trajectory. This definition ensures that r_0 adapts to the actual spread of the particle within each window, providing a data-driven measure of confinement scale.

n. **Velocity autocorrelation function :** This feature measures the degree to which the velocity of a particle at one time is correlated with its velocity at a different time. This indicates the nature of the motion of a particle and distinguish them. Here we calculate the velocity autocorrelation function (VACF) for lag 1, thus comparing the velocities between consecutive time steps **(Eq.S31)**:

$$VACF\ (n = 1) = \frac{1}{N-2}\sum_{k=1}^{N-2}(v_{i+1} \cdot v_i) \tag{S31}$$

where:

v_i is the velocity vector at time step i,

v_{i+1} is the velocity vector for next time step $i + 1$

N is the total number of time steps in a trajectory.

Higher or positive value suggests that the particle retains its direction, indicating DM, while lower or negative value suggests random or ND.

3. Computational Details

All computations were performed using MATLAB 2023a on a Windows 10 operating system. The system was equipped with an Intel(R) Core(TM) i7-10700T CPU running at 2.00 GHz (1.99 GHz base frequency).

4. Movie Simulations

We simulated the realistic dynamics of single emitters diffusing in an aqueous solution to generate ground truth trajectories representative of typical diffusion behaviors. These trajectories were then used as input for 3D dynamics simulations, allowing us to create synthetic fluorescence microscopy movies. This workflow enabled us to assess the model's accuracy in a conventional experimental analysis pipeline, bridging ideal simulations and practical imaging conditions. All simulation parameters were consistent with those listed in Table S1, except for the number of particles (17) and trajectory lengths.

4.1. 3D Dynamics Simulations

Simulations are needed to record ground truth of the trajectory data for quantifying the 3D-SPT classifier accuracy. These simulations are based on scalar diffraction theory and Fourier

optics. The image of an isotropic point is computed based on the parameters of the optical system, where, light propagation through a circular aperture produces well-defined Airy disk point spread function, or Airy PSF. [9]The pupil function is calculated and sampled explicitly as a function of the numerical aperture (NA), wavelength of the emission, and the effective pixel size of a detector.

The double-helix PSF is a well-established PSF model characterized by having two lobes that rotate around a center point when defocus is applied. DH phase mask is generated using Gauss-Laguerre modes weighted at 9 different points of defocus.[10] This mask is automatically resized to match or slightly exceed the pupil function to reduce unmodulated light at the image plane. [11,12]

The phase pattern is applied at the back focal plane of the simulated optical system. Practically, researchers utilize 4f systems composed of two plano-convex lenses, where the phase pattern is positioned at the conjugate back focal plane (the Fourier plane) using an etched, dielectric phase mask or a programmed liquid crystal spatial light modulator (SLM). The defocus effect is applied by calculating the phase shift as a function of the optical path difference. [13] The values of defocus phase are also spatially constrained by the pupil function. Conveniently, the Airy disk PSF can be determined using the conventional discrete Fourier transform (FFT) of the pupil function. The simultaneous convolution of the pupil function, defocus phase, and phase mask results in the z-dependent DH PSF at the image plane.[14,15]

Once the image of the emitter is generated, the intensity is normalized to unity and the emitter is cropped and positioned onto an array of pixels of user-defined dimensions. The x,y position of the cropped PSF within the array is a direct application of the 2D coordinate given in the trajectory file, scaled by the resolution defined by the optical system.

Finally, white noise and dark noise are applied to the resulting image. The white noise is given as a Poisson distribution based on the number of signal photons defined by the user. The dark noise is modeled from the specifications of a Teledyne Photometrics Prime 95b sCMOS camera, which has been used recently in the research group for single molecule tracking experiments at chromatographic interfaces. [16]Additionally, a tunable signal parameter allows the researcher to simulate the low signal-to-noise conditions under which single-experiments are frequently conducted. A peak intensity of approximately 150 counts is utilized, based on calibration experiments with fluorescent polystyrene beads with the Photometrics camera. The PSF simulation process is repeated for each emitter and each frame of the trajectory data, until all frames are completed. The resulting movie is stored as a *uint16* .tif file.

Note on Localization Errors: While our simulation framework does not explicitly define localization errors, it realistically incorporates static localization effects through the end-to-end process of PSF-based image simulation and tracking. The use of a scalar diffraction model, DH-PSF convolution, and the KNOT algorithm introduces sub-pixel localization uncertainty, anisotropic resolution (especially in Z), and trajectory fragmentation. Dynamic localization error (motion blur) is not included in the current simulations, as each movie frame represents an instantaneous snapshot without temporal integration over exposure. These artifacts reflect practical imaging limitations in real experiments, such as noise, optical blurring, and linking errors. This approach ensures that the extracted trajectories realistically capture localization challenges encountered during 3D single particle tracking without requiring explicit noise modeling.

4.2. Recovering Trajectory Data from Simulated Movies

The simulated 3D dynamics movies are then analyzed by KNOT[17], an unbiased tracking algorithm previously developed in the lab. KNOT utilizes the alternating direction method of multipliers (ADMM) algorithm to retrieve the "experimental" trajectories from simulated movie data. The KNOT-retrieved trajectories are finally analyzed by the deep-learning network to characterize different types of diffusion.

5. Extracted Trajectory Data Analysis

The KNOT particle tracking algorithm is used to extract trajectories from noisy movie frames, generating a dataset of 3D spatial coordinates $[x, y, z]$ along a time sequence t.

5.1. Calibration of Tracked Trajectories

Since the KNOT algorithm outputs trajectories in voxel units, they must be converted into real-world microns for proper analysis. The spatial calibration for the X and Y coordinates involves shifting the values relative to the imaging plane center. This is done by subtracting 256/2 from the raw coordinates and negating them to align with the correct coordinate system. After re-centering, a scaling factor of 0.06875 is applied to convert voxel units into microns.

The Z-axis calibration requires a different approach since depth values in imaging systems do not follow the same scaling as X and Y. Instead, a linear calibration equation is used:

$$Z_{microns} = \frac{Z_{raw} - y_{intercept}}{slope} \quad \text{(S32)}$$

where, $y_{intercept}$ and $slope$ are predetermined system parameters from experimental calibration.

5.2. Filtering of Invalid and Noisy Trajectories

To reduce errors from noisy or unreliable trajectories, we applied several filtering steps. Trajectories containing only zero values were discarded, as were those shorter than the window length (WinLen = 29 frames), which lack sufficient data for classification. Disconnected trajectories with gaps larger than one frame were also removed, while small gaps (≤1 frame) were interpolated. These filtering procedures help mitigate the impact of tracking errors and localization noise, particularly along the Z-axis, where precision is typically lower in 3D imaging systems.

5.2. Trajectory Matching Using Least-Squares Optimization

The filtered trajectories were then matched with their corresponding ground truth trajectories. This is done implementing least-squares approach to minimize deviations between them. The tracked trajectories were compared against all ground truth trajectories by calculating sum of deviations:

$$d = \sum (T_{adjusted} - T_{tracked})^2 \quad \text{(S33)}$$

where $T_{adjusted}$ is the ground truth trajectories and $T_{tracked}$ is the extracted trajectory. The trajectory with the smallest squared deviation is selected as the closest match. This method ensures

that each extracted trajectory is paired with correct reference trajectory, thereby reducing misalignment errors.

6. Experimental Validation of Normal Diffusion

a. **Coverslip cleaning:** Microscope coverslips (No. 1; VWR, 22 × 22 mm) are sonicated in a Liquinox detergent solution for 30 minutes, followed by sequential 15-minute sonication steps in deionized (DI) water, methanol, and acetone. Afterwards, the coverslips are rinsed in a base piranha solution (1:1:5 ratio of 30% NH_4OH, 30% H_2O_2, and H_2O) at 80 °C for 30 minutes. The coverslips are then treated with oxygen plasma (PDC-32G, Harrick Plasma) for 2 minutes.

b. **Microscopy:** Fluorescence imaging is conducted with a custom-built microscope illuminated with a 532 nm laser (SuperK FIANIUM) operating at approximately 3 mW. The excitation beam is collimated onto the sample using a high numerical aperture oil-immersion objective (100×, NA = 1.46; Carl Zeiss, alpha Plan-Apochromat) in epifluorescence geometry. The emission is then reflected by a Chroma z532/633rpc dichroic mirror and focused by a tube (f = 165 mm). The emission propagates through a 4f optical system incorporating two lenses (f = 100 mm) and a Double Helix (DH) phase mask (Double Helix LLC). The resulting image is captured using a back-illuminated sCMOS camera (Photometrics Prime 95B).

c. **Single-Particle Measurements:** Fluorescent polystyrene beads (0.1 μm, orange FluoSpheres, Thermo Fisher Scientific) are gently shaken before being diluted to a 100,000x concentration in DI water. A volume of 25 μL of the fluorescent beads is pipetted onto the surface of the coverslip and allowed to settle for 1 minute while positioned over the objective. After settling, movies containing 1000 images are recorded with an

integration time of 30 milliseconds. An unbiased tracking algorithm – KNOT is employed to analyze the dynamics of the fluorescent beads.[17]

d. **Classification Results:** The experimental trajectories of 0.1 μm fluorescent polystyrene beads diffusing in 40% glycerol were analyzed using our optimized model. As expected under these conditions (theoretical D = 0.2 $\mu m^2/s$), most trajectories (85.7%) were classified as ND, consistent with free diffusion in viscous solution. Smaller fractions were DM (5.7%), DM+ND (2.9%), and ND+CD (5.7%), with no trajectories classified as purely AD, CD, or other combinations. These few misclassifications are likely due to brief surface contacts, slight drift, or noise, common in experiments. Figure S12 shows the distribution of classified motion types. This analysis shows that the model correctly identifies ND as the main motion type, matching the expected diffusion coefficient, while still detecting minor experimental artifacts, supporting its use for real single-particle tracking data.

7. Supporting Figures

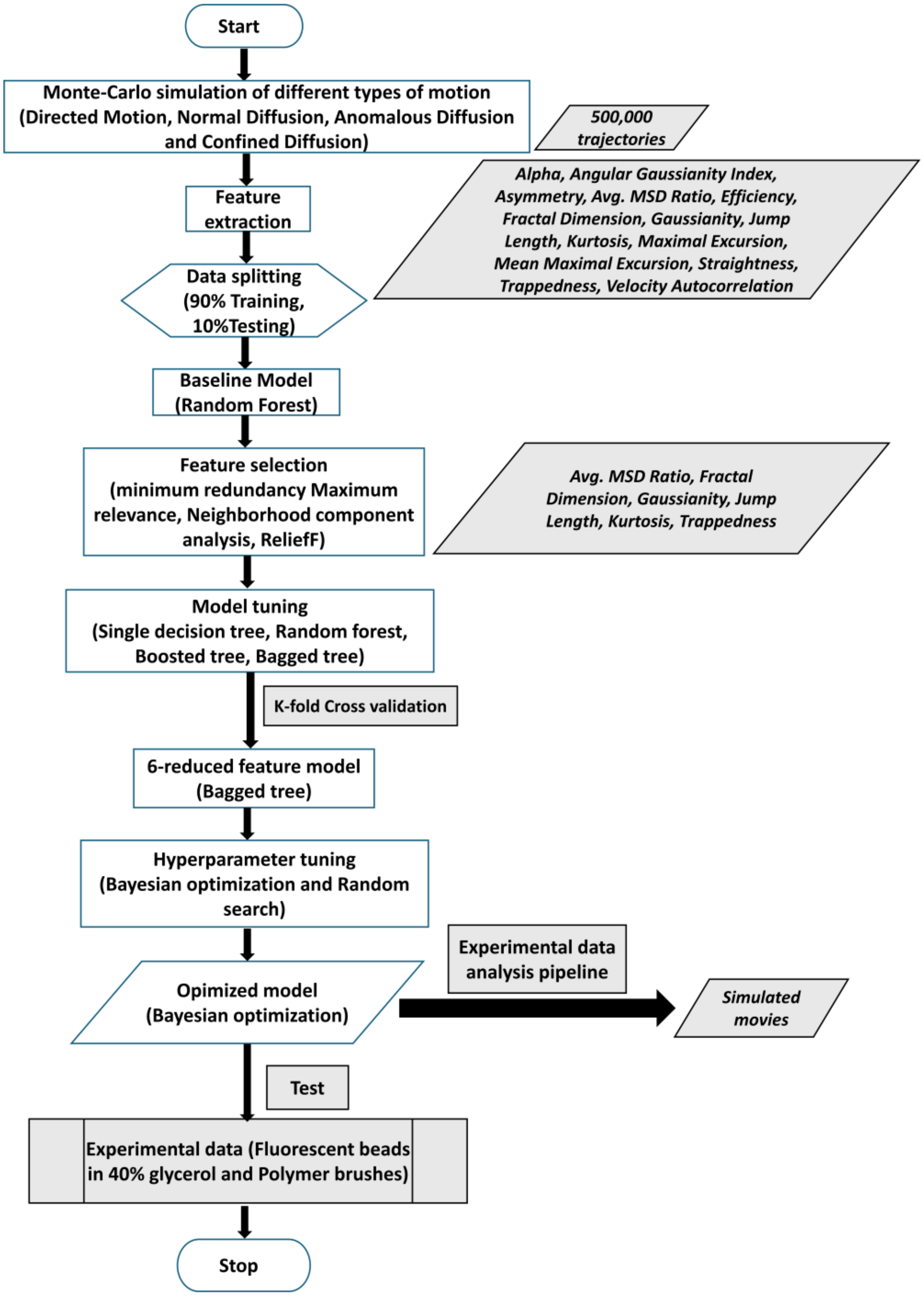

Figure S1: Flowchart illustrating the machine learning pipeline to classify different types of motion using Monte-Carlo simulations. The process begins with simulating 500,000 trajectories (DM, ND, AD, and CD). Key features are extracted, followed by data splitting into training (90%) and testing (10%). A baseline Random Forest model is established. Feature selection reduces the dataset to six main features. Various models are tuned using K-fold Cross Validation, leading to a reduced feature Bagged Tree model. Hyperparameter tuning (Bayesian optimization and

Random search) refines the model, resulting in an optimized model via Bayesian optimization. The final model is tested using experimental data (fluorescent beads on 40% glycerol and polymer brushes) and is also implemented to generate a pipeline for classification of trajectories from movies.

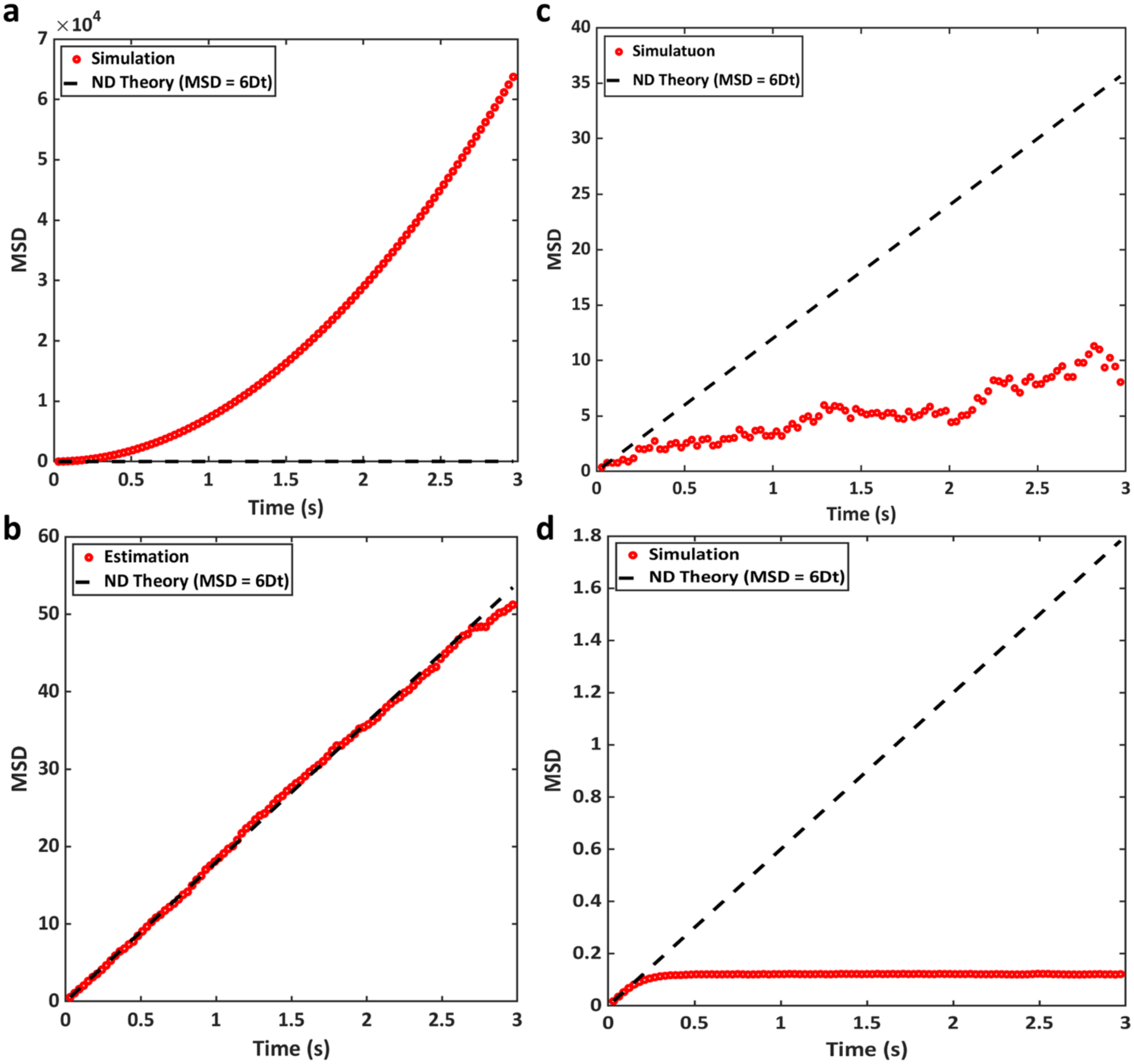

Figure S2: Comparison of mean squared displacement (MSD) versus time for different motion types. The red circles indicate simulated or estimated MSD values, while the black dashed lines represent the theoretical MSD predictions using the ND model (MSD = 6Dt). (a) DM shows accelerated MSD growth relative to the theoretical linear trend. (b) ND closely follows the expected linear MSD behavior. (c) AD displays sublinear MSD growth, deviating from the linear theory. (d) CD exhibits MSD saturation over time, consistent with restricted spatial displacement.

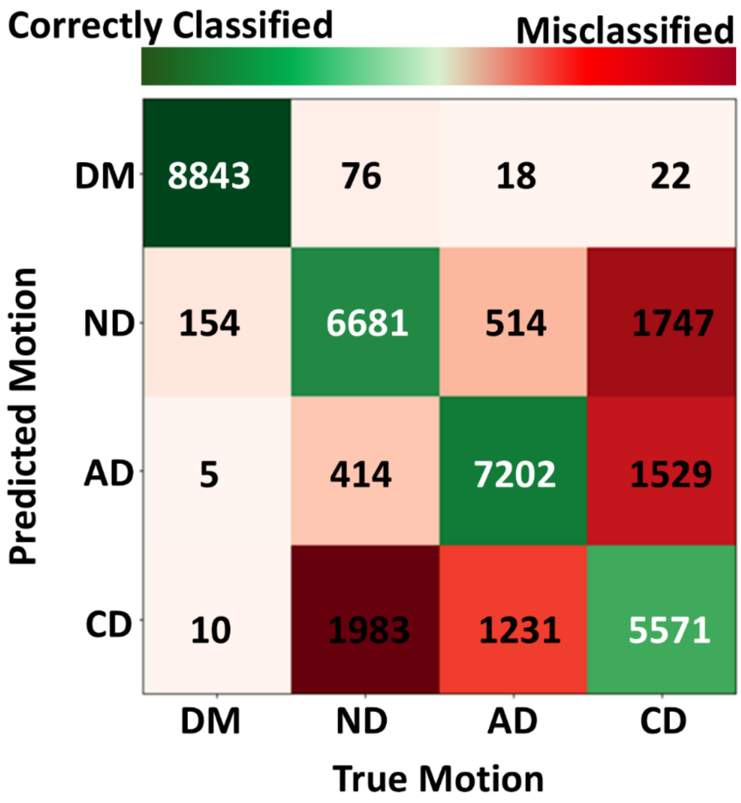

Figure S3: Confusion matrix for the baseline model (accuracy = 78%) shows classification performance across four motion types. Each cell indicates the number of predicted instances (rows) versus true motion types (columns). Correct classifications appear along the diagonal (green), while off-diagonal entries (red) represent misclassifications. The color gradient emphasizes classification correctness, with green indicating correct predictions and red indicating errors.

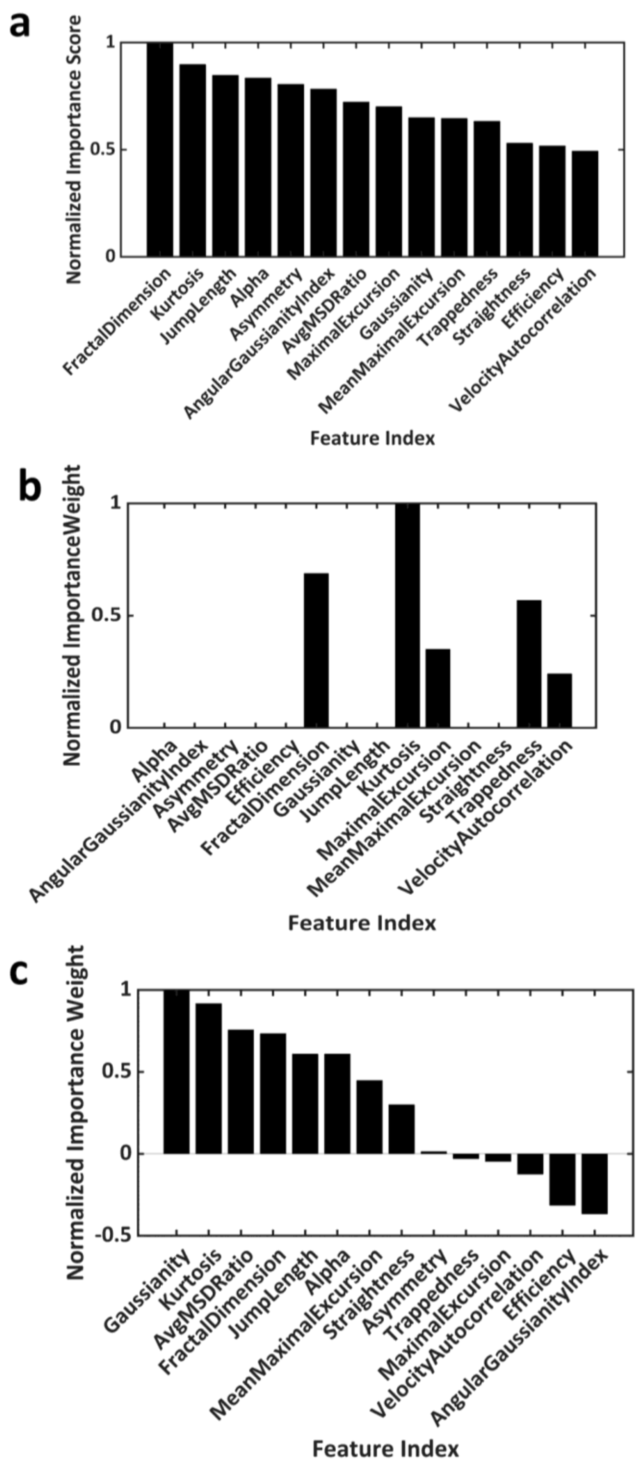

Figure S4: The three feature selection algorithms identify important features that contribute most to the motion classification in 3D. (a) Minimum Redundancy Maximum Relevance (mRMR) algorithm, which shows Fractal Dimension, Kurtosis, and Jump length as the top 3 features; (b) Neighborhood Component Analysis (NCA) algorithm, which highlights Kurtosis, Fractal Dimension, and Trappedness as the top 3 features; and (c) ReliefF algorithm, which identifies Gaussianity, Kurtosis and Average MSD Ratio as the top 3 features. Each bar represents the normalized importance score for a specific feature, indicating its relevance in the motion classification model.

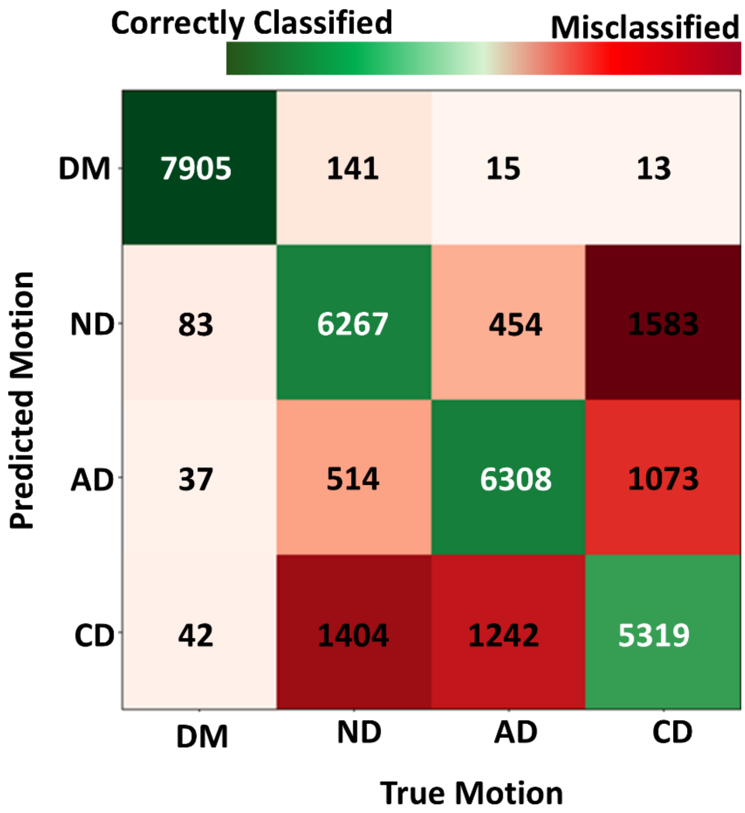

Figure S5: Confusion matrix showing the classification performance of the tuned bagged ensemble model (79% accuracy) on simulated 3D trajectories. The rows represent the predicted motion classes and the columns represent the true motion classes. Green shading indicates correct classifications, while red shading highlights misclassifications. The matrix illustrates strong performance for DM and AD, with most errors arising from confusion between ND and CD.

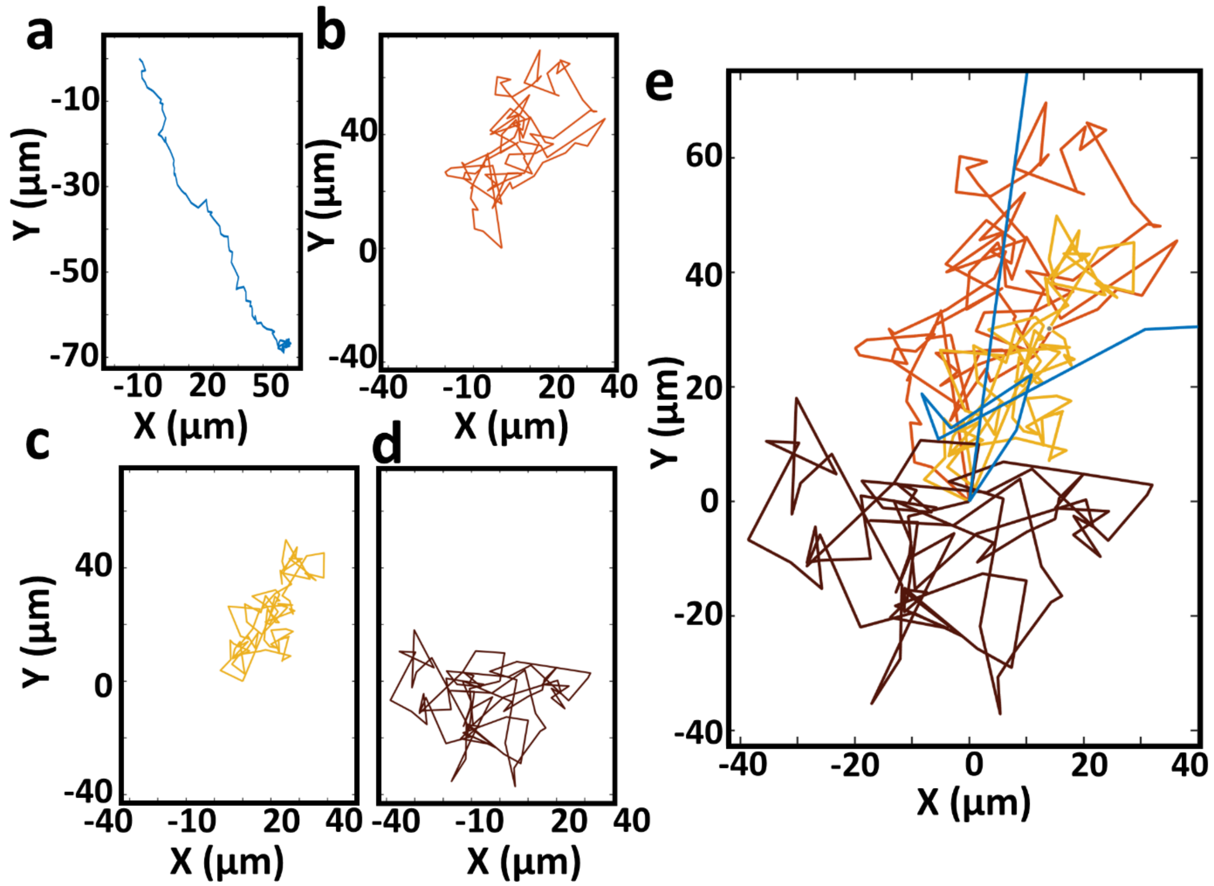

Figure S6: Representative 2D projections of simulated trajectories for each motion type: (a) DM, (b) ND, (c) AD, (d) CD, and (e) mixed motion. The trajectories were generated by removing the Z-dimension from 3D data, preserving temporal structure and motion labels while reducing spatial information. The plots illustrate the distinct patterns associated with each motion type in 2D space.

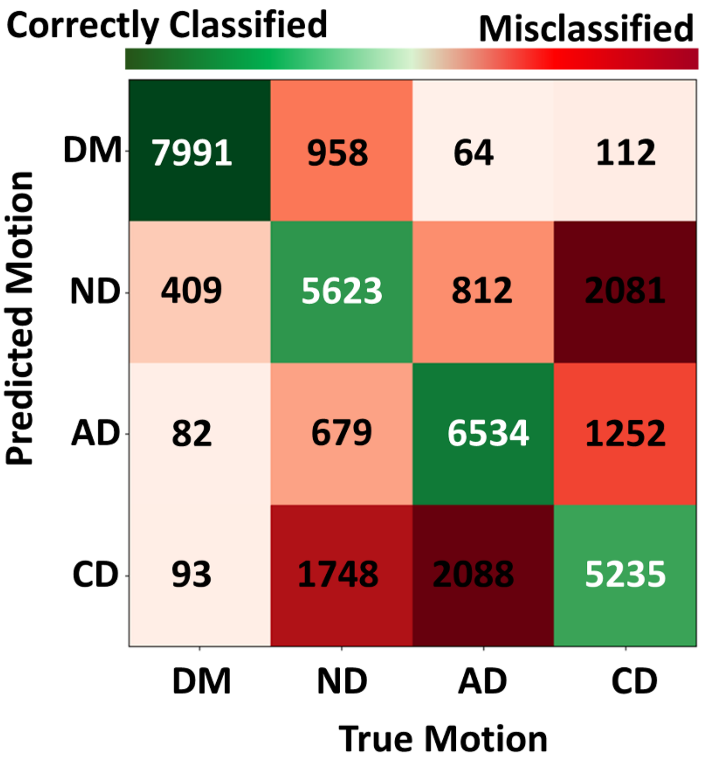

Figure S7: Confusion matrix showing the classification performance of the baseline model on projected 2D trajectories (overall accuracy: 71%). Each cell indicates the number of sub-trajectories where the true motion type was classified as the predicted motion type. Correct classifications appear along the diagonal in green, while misclassifications are shaded red. The matrix illustrates that DM was classified most accurately, while CD showed the greatest overlap with ND and AD.

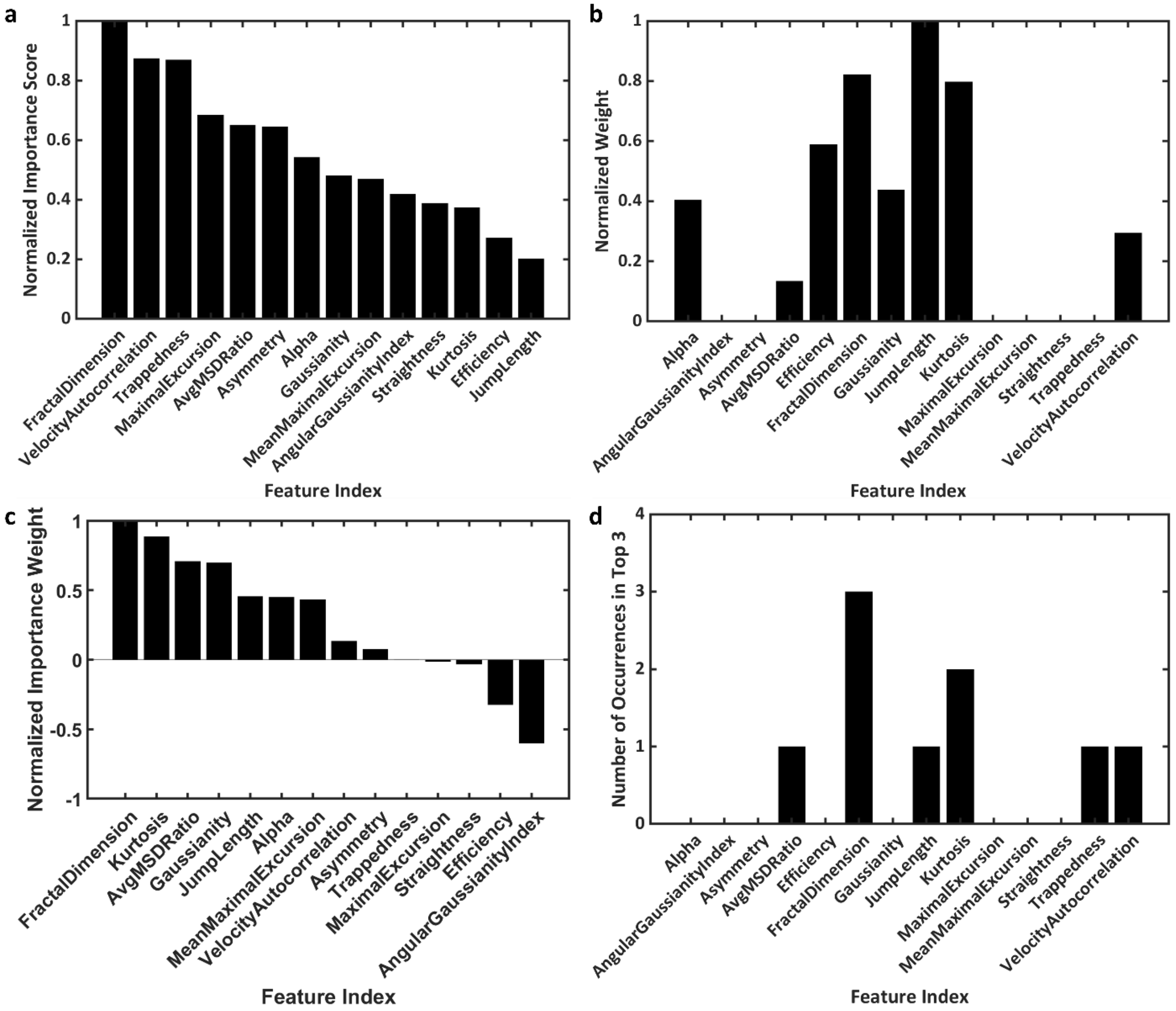

Figure S8: The three feature selection algorithms identify important features that contribute most to motion classification in projected 2D trajectories. (a) Minimum Redundancy Maximum Relevance (mRMR) algorithm shows Fractal Dimension, Velocity Autocorrelation, and Trappedness as the top 3 features. (b) Neighborhood Component Analysis (NCA) highlights Kurtosis, Gaussianity, and Jump Length as the top 3 features. (c) ReliefF identifies Fractal Dimension, Avg MSD Ratio, and Kurtosis as the top 3 features. (d) The bar chart summarizes the number of times each feature appeared in the top three across all methods. Each bar represents the normalized importance score or frequency of selection, indicating the feature's relevance in the classification model.

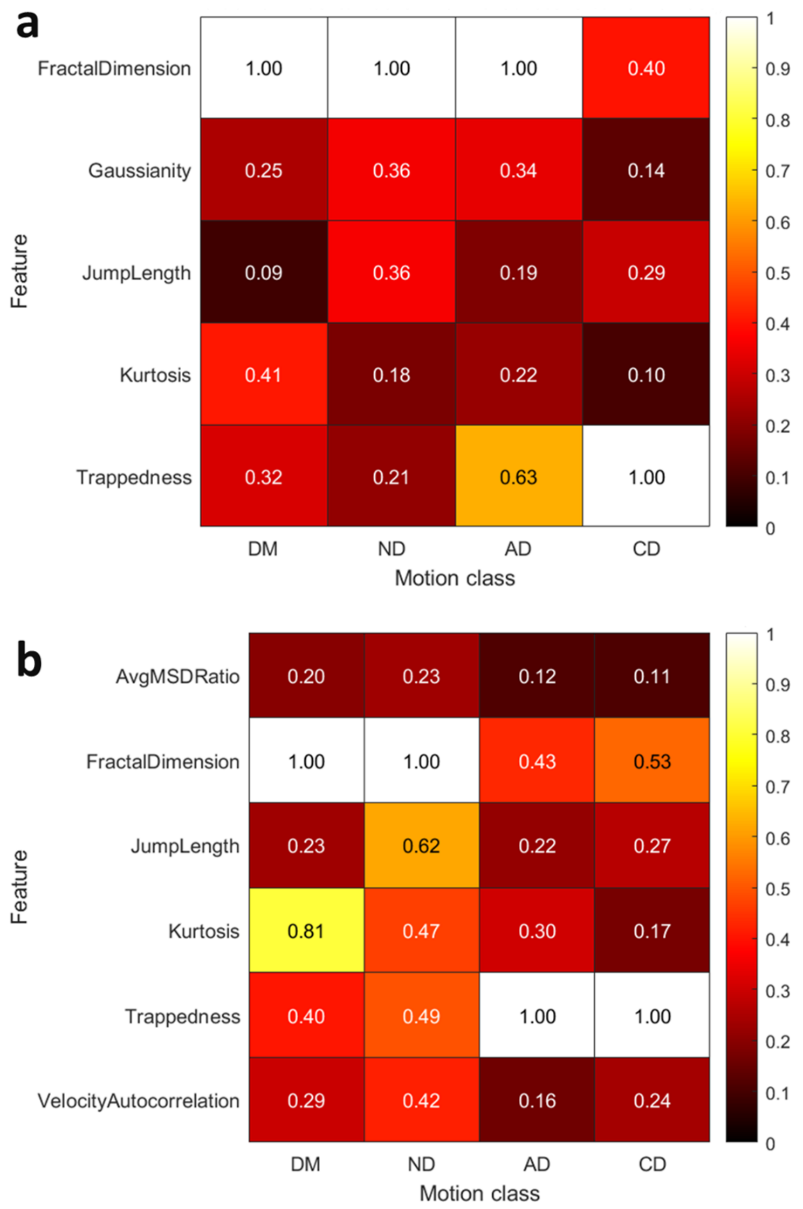

Figure S9: Class-wise permutation importance heatmaps show normalized feature importance (0 to 1) for identifying DM, ND, AD, and CD. (a) 3D, where Fractal Dimension is consistently the strongest feature for DM, ND, and AD (1.00), while Trappedness dominates CD (1.00) and supports AD (0.63). Other features like Kurtosis and Jump Length play smaller roles.These results highlight that Fractal Dimension and Trappedness are robust across dimensions, while features like Kurtosis and Velocity Autocorrelation shift in relevance depending on spatial context; and (b) Projected 2D, where Fractal Dimension is the top feature for DM and ND (1.00) and contributes moderately to AD (0.43) and CD (0.53). Kurtosis is most important for DM (0.81), while Jump Length aids ND classification (0.62). Trappedness is key for AD and CD (both 1.00). Velocity Autocorrelation contributes modestly to DM (0.29) and ND (0.42)

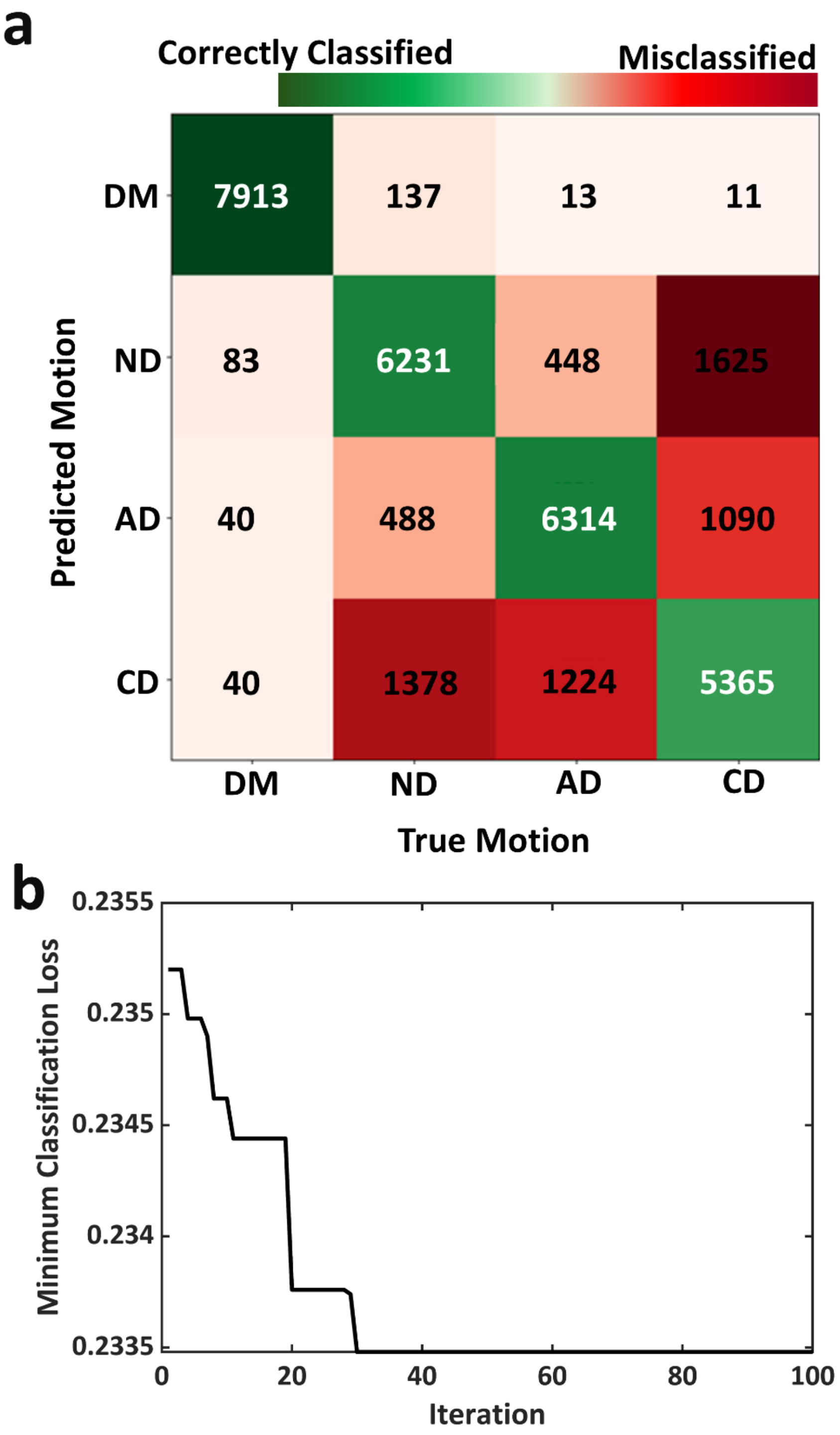

Figure S10: The optimized ensemble model accurately classifies most motion types, with particularly strong performance on DM and AD, and stable convergence of the hyperparameter tuning process. (a) Confusion matrix showing the classification performance of the hyperparameter-optimized bagged ensemble model. The color scale reflects correct classifications (green) and misclassifications (red). DM was classified with the highest accuracy, followed by AD, ND, and CD. (b) Convergence trace of Bayesian hyperparameter optimization on a subset of 50,000 training trajectories, demonstrating a steady decrease in cross-validated classification loss over ~30 iterations before plateauing, indicating stable convergence.

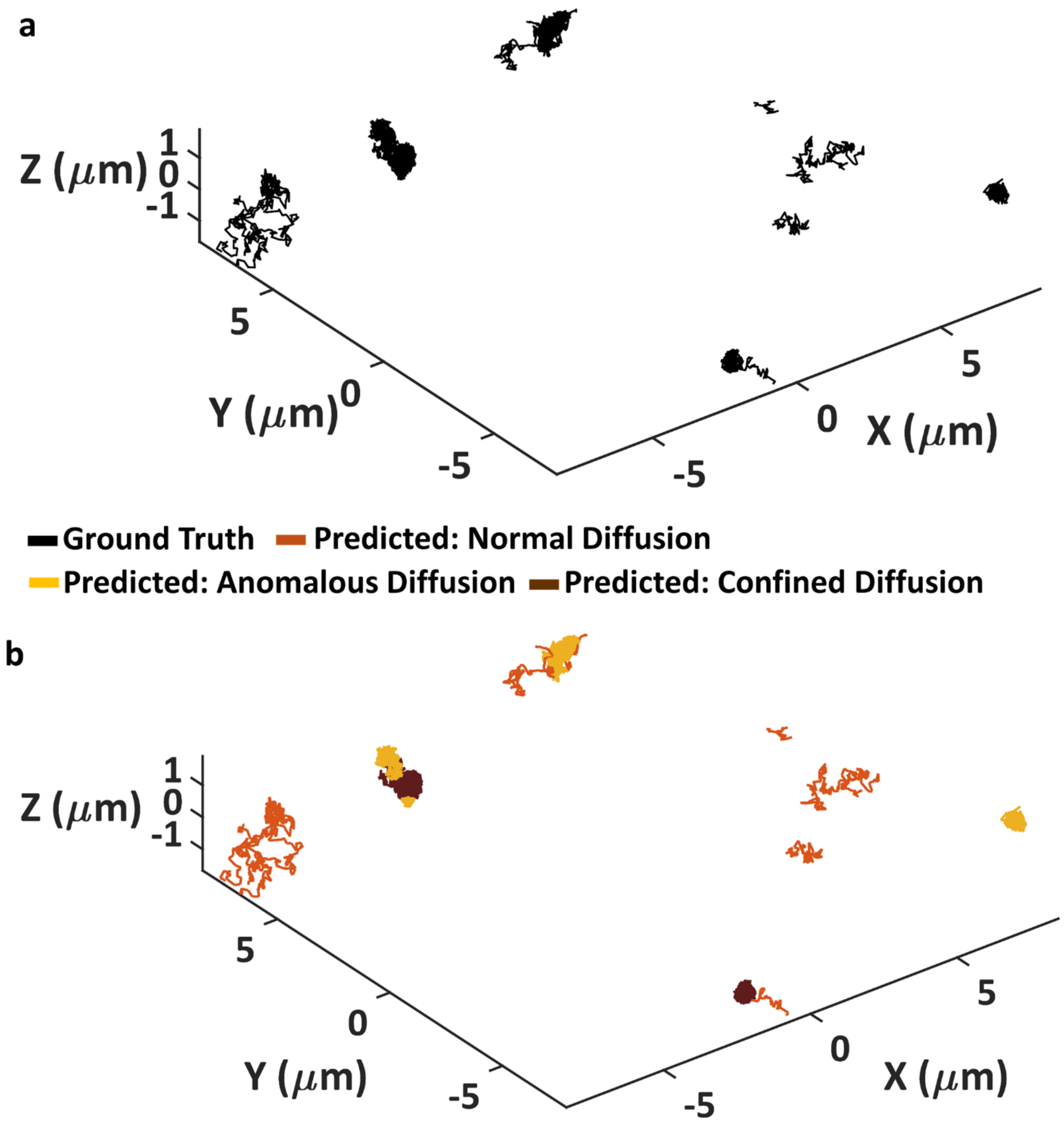

Figure S11: Classification of different types of motion in simulated movies mimicking experimental conditions using the optimized model. (a) Particle trajectories are extracted from the simulated movies having combination of ND,AD and CD in both two and three motion type combinations using the KNOT particle tracking algorithm,[17] producing time-sequenced spatial coordinates$[x, y, z]$ over time t. (b) The extracted trajectories are processed through the calibration and filtering procedures followed by feature extraction to characterize the motion dynamics. The optimized model classifies ND, AD, or CD based on learned features and are visualized in 3D, with trajectories color-coded by their predicted motion type.

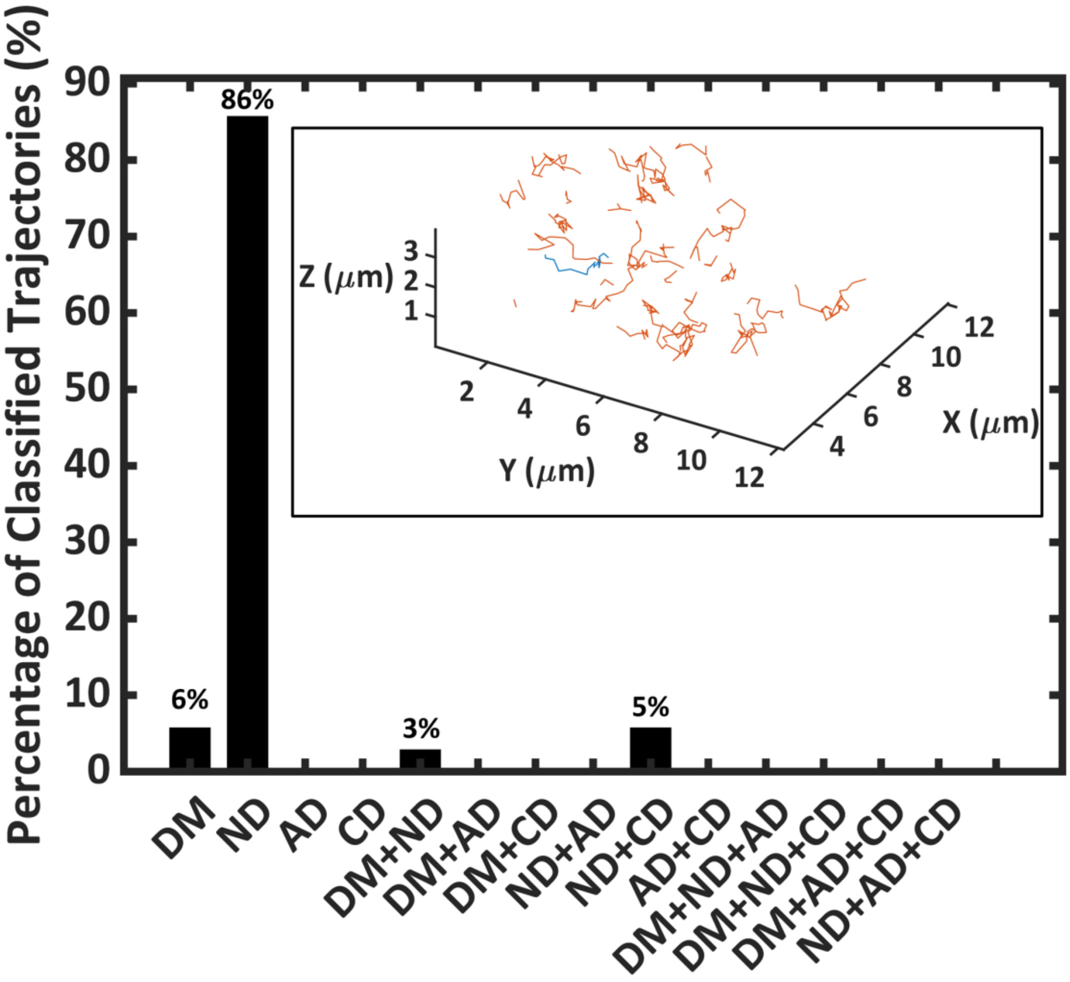

Figure S12: The majority of experimental trajectories of 0.1 µm fluorescent polystyrene beads diffusing in 40% glycerol were classified as ND by the optimized model. A large majority of trajectories (86%) were assigned to ND, with smaller proportions identified as DM, (6%), mixed categories (DM+ND, 3%; ND+CD, 6%), and none as AD, CD, or other combinations. The inset shows representative 3D trajectories, color-coded by motion type.

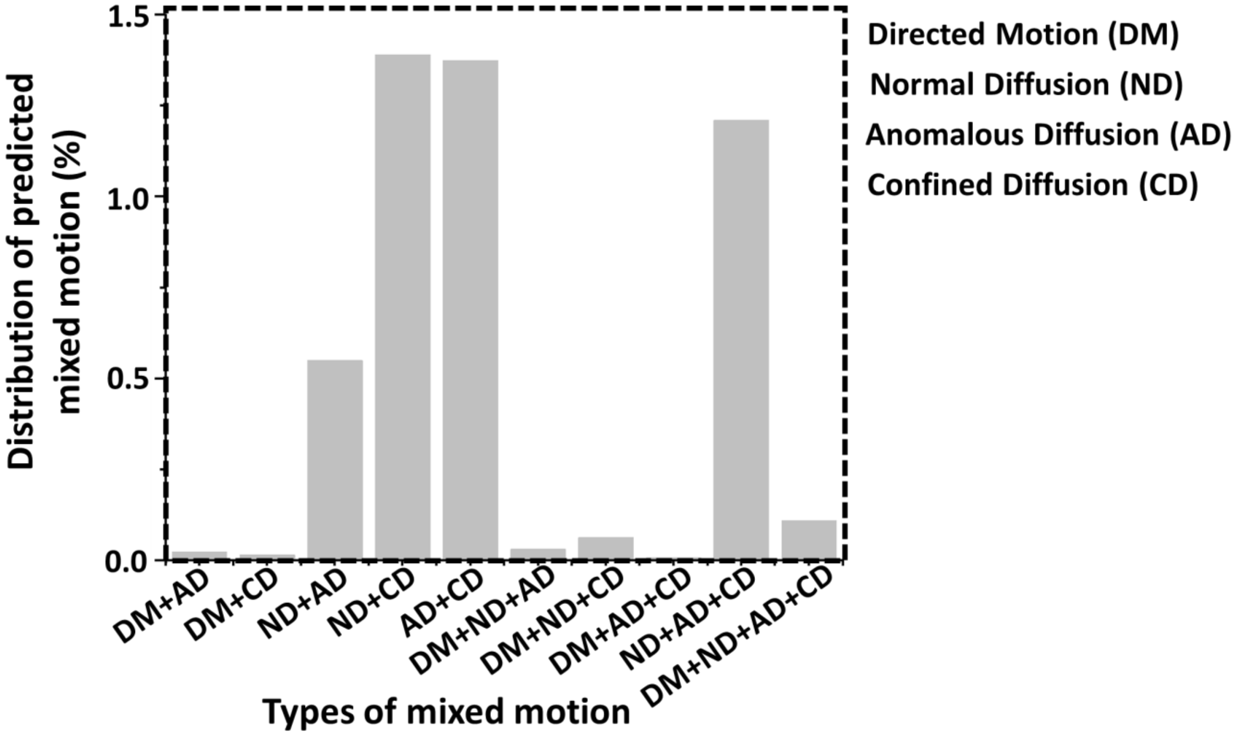

Figure S13: 5% of probe molecules in the polymer brush system exhibit mixed motion behavior. The histogram, derived from the experimental data of Fan et al. (2023),[16] quantifies the distribution of these mixed motion trajectories, capturing combinations among DM, ND, AD and CD.

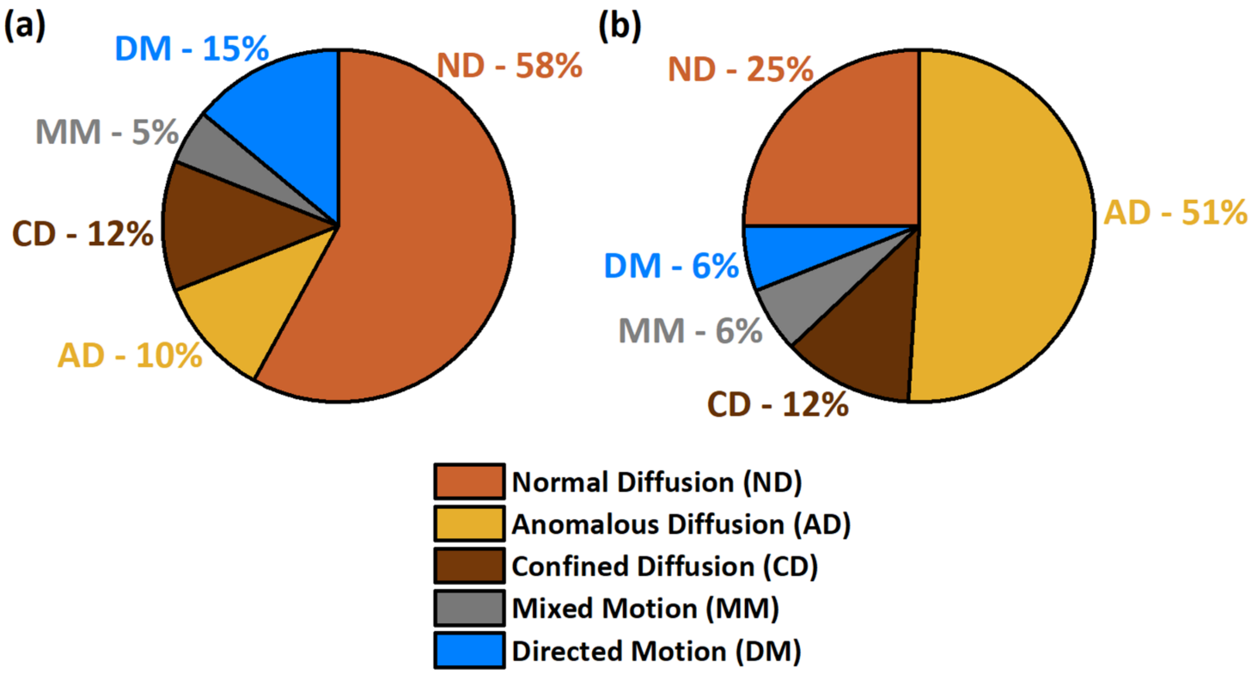

Figure S14: Using our optimized machine learning model, we reanalyzed the previously published data from *Fan et al. (2023)*[16] and uncovered greater heterogeneity in the motion behaviors originally categorized as "unconfined" or "confined." (a) Among trajectories originally labeled "unconfined" (77%), 73% are now classified as ND (58%) or DM (15%), with the remainder showing CD (12%) or MM (5%), highlighting local confinement and directional effects. (b) Of the 23% originally labeled "confined," most are now classified as AD (51%) or ND (25%), indicating dynamics shaped by complex local interactions rather than simple trapping.

8. Supporting Tables

Table S1: Simulation parameters that were used for generating mixed motion. The key variables and their values are detailed.

PARAMETER	DESCRIPTION	VALUE
**T**	Number of frames	100
**P**	Number of particles	5000
**D_MIN**	Minimum diffusion coefficient	0.01μm/s^2
**D_MAX**	Maximum dissusion coefficient	15 μm/s^2
**TIME (DT)**	Time step	0.03 s
**WINLEN**	Window length for trajectory analysis	29
**MINLEN**	Minimum segment length for motion transistions	20
**ALPHA_MIN**	Minimum AD scaling exponent	0.1
**ALPHA_MAX**	Maximum AD scaling exponent	0.7
**B_MIN**	Minimum confinement parameter	0.2
**BMAX**	Maximum confinement parameter	1.2
**KDM1**	DM minimum velocity factor	5
**KDM2**	DM maximum velocity factor	100

Table S2: Bagged trees achieved the highest accuracy and lowest error among the models tested. The table summarizes the accuracy and loss/error for different decision tree models implemented using the six selected features.

MODEL	ACCURACY (%)	LOSS / ERROR
**SINGLE DECISION TREE**	72%	0.28
**RANDOM FOREST**	77%	0.23
**BOOSTED TREES**	73%	0.27
**BAGGED TREES**	79%	0.21

References

(1) Wagner, T.; Kroll, A.; Haramagatti, C. R.; Lipinski, H. G.; Wiemann, M. Classification and Segmentation of Nanoparticle Diffusion Trajectories in Cellular Micro Environments. *PLoS One* **2017**, *12* (1), e0170165. https://doi.org/10.1371/journal.pone.0170165.

(2) Seckler, H.; Szwabiński, J.; Metzler, R. Machine-Learning Solutions for the Analysis of Single-Particle Diffusion Trajectories. *J. Phys. Chem. Lett.* **2023**, *14* (35), 7910–7923. https://doi.org/10.1021/acs.jpclett.3c01351.

(3) Tyler, D. E. Statistical Analysis for the Angular Central Gaussian Distribution on the Sphere. *Biometrika* **1987**, *74* (3), 579–589. https://doi.org/10.1093/biomet/74.3.579.

(4) Saxton, M. J. Lateral Diffusion in an Archipelago. Single-Particle Diffusion. *Biophys. J.* **1993**, *64* (6), 1766–1780. https://doi.org/10.1016/S0006-3495(93)81548-0.

(5) Katz, M. J.; George, E. B. Fractals and the Analysis of Growth Paths. *Bull. Math. Biol.* **1985**, *47* (2), 273–286. https://doi.org/10.1007/BF02460036.

(6) Ernst, D.; Köhler, J.; Weiss, M. Probing the Type of Anomalous Diffusion with Single-Particle Tracking. *Phys. Chem. Chem. Phys.* **2014**, *16* (17), 7686–7691. https://doi.org/10.1039/c4cp00292j.

(7) Saxton, M. J.; Jacobson, K. Single-Particle Tracking: Applications to Membrane Dynamics. *Annu. Rev. Biophys. Biomol. Struct.* **1997**, *26*, 373–399. https://doi.org/10.1146/annurev.biophys.26.1.373.

(8) Helmuth, J. A.; Burckhardt, C. J.; Koumoutsakos, P.; Greber, U. F.; Sbalzarini, I. F. A Novel Supervised Trajectory Segmentation Algorithm Identifies Distinct Types of Human Adenovirus Motion in Host Cells. *J. Struct. Biol.* **2007**, *159* (3), 347–358. https://doi.org/10.1016/j.jsb.2007.04.003.

(9) Goodman, J. W. *Introduction to Fourier Optics*; McGraw-Hill: New York, 1969.

(10) Schechner, Y. Y.; Shamir, J. Propagation-Invariant Wave Fields. *J. Opt. Soc. Am. A* **2000**, *17* (2), 294–303.

(11) Pavani, S. R. P.; Piestun, R. High-Efficiency Rotating Point Spread Functions. *Opt. Express* **2008**, *16* (5), 3484–3489. https://doi.org/10.1364/oe.16.003484.

(12) Gustavsson, A. K.; Petrov, P. N.; Lee, M. Y.; Shechtman, Y.; Moerner, W. E. 3D Single-Molecule Super-Resolution Microscopy with a Tilted Light Sheet. *Nat. Commun.* **2018**, *9* (1), 123. https://doi.org/10.1038/s41467-017-02563-4.

(13) Stokseth, P. A. Properties of a Defocused Optical System. *J. Opt. Soc. Am.* **1969**, *59* (10), 1314–1324. https://doi.org/10.1364/josa.59.001314.

(14) Wang, W.; Ye, F.; Shen, H.; Moringo, N. A.; Dutta, C.; Robinson, J. T.; Landes, C. F. Generalized Method to Design Phase Masks for 3D Super-Resolution Microscopy. *Opt. Express* **2019**, *27* (3), 3799–3810. https://doi.org/10.1364/oe.27.003799.

(15) Jesacher, A.; Booth, M. J. Parallel Direct Laser Writing in Three Dimensions with Spatially Dependent Aberration Correction. *Opt. Express* **2010**, *18* (20), 21090–21099. https://doi.org/10.1364/oe.18.021090.

(16) Fan, D.; Bajgiran, S. R.; Samghabadi, F. S.; Dutta, C.; Gillett, E.; Rossky, P. J.; Conrad, J. C.; Marciel, A. B.; Landes, C. F. Imaging Heterogeneous 3D Dynamics of Individual Solutes in a Polyelectrolyte Brush. *Langmuir* **2023**, *39* (24), 8532–8539. https://doi.org/10.1021/acs.langmuir.3c00868.

(17) Zepeda O, J.; Bishop, L. D. C.; Dutta, C.; Sarkar-Banerjee, S.; Leung, W. W.; Landes, C. F. Untying the Gordian KNOT: Unbiased Single Particle Tracking Using Point Clouds and Adaptive Motion Analysis. *J. Phys. Chem. A* **2021**, *125* (39), 8723–8733. https://doi.org/10.1021/acs.jpca.1c06100.
